# Trustworthy deep learning framework for the detection of abnormalities in X-ray shoulder images

**DOI:** 10.1371/journal.pone.0299545

**Published:** 2024-03-11

**Authors:** Laith Alzubaidi, Asma Salhi, Mohammed A.Fadhel, Jinshuai Bai, Freek Hollman, Kristine Italia, Roberto Pareyon, A. S. Albahri, Chun Ouyang, Jose Santamaría, Kenneth Cutbush, Ashish Gupta, Amin Abbosh, Yuantong Gu

**Affiliations:** 1 School of Mechanical, Medical, and Process Engineering, Queensland University of Technology, Brisbane, QLD, Australia; 2 Queensland Unit for Advanced Shoulder Research (QUASR)/ARC Industrial Transformation Training Centre—Joint Biomechanics, Queensland University of Technology, Brisbane, QLD, Australia; 3 Centre for Data Science, Queensland University of Technology, Brisbane, QLD, Australia; 4 Akunah Medical Technology Pty Ltd Company, Brisbane, QLD, Australia; 5 Technical College, Imam Ja’afar Al-Sadiq University, Baghdad, Iraq; 6 School of Information Systems, Queensland University of Technology, Brisbane, QLD, Australia; 7 Department of Computer Science, University of Jaén, Jaén, Spain; 8 School of Medicine, The University of Queensland, Brisbane, QLD, Australia; 9 Greenslopes Private Hospital, Brisbane, QLD, Australia; 10 School of Information Technology and Electrical Engineering, Brisbane, QLD, Australia; University of Manitoba, CANADA

## Abstract

Musculoskeletal conditions affect an estimated 1.7 billion people worldwide, causing intense pain and disability. These conditions lead to 30 million emergency room visits yearly, and the numbers are only increasing. However, diagnosing musculoskeletal issues can be challenging, especially in emergencies where quick decisions are necessary. Deep learning (DL) has shown promise in various medical applications. However, previous methods had poor performance and a lack of transparency in detecting shoulder abnormalities on X-ray images due to a lack of training data and better representation of features. This often resulted in overfitting, poor generalisation, and potential bias in decision-making. To address these issues, a new trustworthy DL framework has been proposed to detect shoulder abnormalities (such as fractures, deformities, and arthritis) using X-ray images. The framework consists of two parts: same-domain transfer learning (TL) to mitigate imageNet mismatch and feature fusion to reduce error rates and improve trust in the final result. Same-domain TL involves training pre-trained models on a large number of labelled X-ray images from various body parts and fine-tuning them on the target dataset of shoulder X-ray images. Feature fusion combines the extracted features with seven DL models to train several ML classifiers. The proposed framework achieved an excellent accuracy rate of 99.2%, F1_*Score*_ of 99.2%, and Cohen’s kappa of 98.5%. Furthermore, the accuracy of the results was validated using three visualisation tools, including gradient-based class activation heat map (Grad CAM), activation visualisation, and locally interpretable model-independent explanations (LIME). The proposed framework outperformed previous DL methods and three orthopaedic surgeons invited to classify the test set, who obtained an average accuracy of 79.1%. The proposed framework has proven effective and robust, improving generalisation and increasing trust in the final results.

## Introduction

The role of ML in orthopaedic practice is growing incredibly and has become increasingly important [1, 2]. Supporting clinicians in detecting pathological findings on radiographs could be helpful to optimise accuracy. Radiologists and physicians use radiographs in orthopaedic routines to assess bone anatomy and detect joint abnormalities [3]. Imaging evaluation can be challenging, especially in an emergency setting with high-volume care overload [4, 5]. Subsequently, the level of experience in interpreting these images could vary, which could affect the accuracy.

Despite advances in computer vision in recent years, the detection of shoulder joint abnormalities using X-ray imaging remains a challenging task that DL solutions can address more accurately. Specifically, DL algorithms have been proven to be a reliable tool in radiology and orthopaedics to save time and help medical professionals, particularly those less experienced, make an accurate diagnosis [6–10].

However, DL algorithms require a large dataset to improve the learning efficiency of a specific task [11, 12]. This limits the utilisation of DL power in medical imaging applications when a large dataset is unavailable. To overcome data scarcity in the medical field, TL is widely recognised as a powerful technique to tackle the issue [13–16]. TL with a convolutional neural network (CNN) aims to leverage existing generalised knowledge from related source tasks to improve performance on a specific target task with a relatively small dataset [11]. Using CNNs pre-trained on ImageNet, which is the largest publicly available dataset of natural images [17, 18], has become the standard method for TL. However, the fundamental mismatch between medical images and ImageNet in terms of size, features, and tasks makes it unsuitable for TL in medical imaging applications [17, 19]. TL from pre-trained models in the ImageNet dataset has been proven to be ineffective for medical imaging tasks, especially greyscale images such as MRI, CT, and X-ray [19–23]. Azizi et al. [20] conducted experiments on two tasks involving medical images: classifying skin conditions based on images from the digital camera and labelling chest radiographs with multiple labels. They found that using self-supervised learning on ImageNet, followed by additional self-supervised learning using specific unlabelled medical images, significantly improved the accuracy of medical image classification compared to using only TL from IamgeNet labelled images. Raghu, Maithra et al. [19] have shown that using a different domain as a source of TL does not significantly impact the performance of medical imaging tasks. Lightweight models trained from scratch can carry out almost as well as models transferred from ImageNet. Silva, Francisco, et al. [22] explored an alternative approach to using TL from pre-trained models of ImageNet for lung cancer tasks. They trained a feature extractor within the same domain as the final task, using more extensive regions of the lung containing nodules. The results showed that this approach effectively captured relevant information for lung cancer tasks, highlighting the importance of comprehensive approaches for enhanced performance. Jin, Boyang Tom, et al. [23] demonstrated that using TL within the same domain for medical applications is highly effective.

Furthermore, for medical applications, the performance of the TL-based model has been shown to depend on the similarity between the source and target domains [24–28]. Alzubaidi et al. [24] found that using a small number of unlabelled images from the same domain as the target task for TL performs better than using a large number of labelled images from a different domain in medical image applications. This was tested on two medical tasks, skin and breast cancer. It was concluded by [25–27] that self-supervised learning, in which DL models are pre-trained using large unlabelled datasets from the same domain, can enhance the performance of downstream tasks. This is particularly useful for training models to perform medical diagnosis tasks, where it can be not easy to obtain large-labelled datasets.

With an increase in publicly available medical imaging datasets, the number of studies that use the TL technique for medical image classification has increased significantly [29]. For the detection of musculoskeletal abnormalities (MSK), Rajpurkar et al. [4] introduced a large dataset of X-ray images of seven different joints of the upper extremities (e.g., elbow, finger, forearm, hand, humerus, shoulder, and wrist) and made them publicly available for research purposes. The MURA dataset contains 40,561 X-ray images labelled negative (normal) and positive (abnormal), including a subset of 8,942 shoulder X-ray images.

Recently, many studies have worked on the MURA dataset with the help of TL configurations, and most CNN models have demonstrated handling the MSK abnormalities detection task [30–32]. However, many of these studies used CNN models pre-trained on ImageNet, which decreased their performance due to its irrelevant features. Furthermore, some studies used ensemble techniques to improve shoulder abnormality detection performance [30, 32]. It is necessary to provide representative features to train ML classifiers. Otherwise, the performance of ML classifiers is poor. Feature fusion is crucial in DL as it allows neural networks to combine and integrate information from multiple sources or layers, permitting them to capture complex patterns and relationships within the data [33, 34]. It enhances the model’s ability to make more accurate and robust predictions across various tasks, ultimately improving the performance and generalisation of DL models [35]. Moreover, it is essential to address the problem of data scarcity before utilising feature fusion techniques [36]. Lastly, most studies on the detection of shoulder abnormalities have not evaluated the performance of the models used to explain the “black box” of DL. The lack of interpretability of the model using the black box is considered a significant barrier to clinical trust and adoption [37–41]. Explaining the black box of DL is critical to detect any bias and make the DL application trustworthy.

In summary, it is necessary to investigate different TL configurations for the shoulder abnormality detection task and propose a TL that can alleviate the domain mismatch problem. For further improvement, the fusion technique could be employed as an efficient method to combine the extracted features by different CNN models. Furthermore, it is essential to support the decision result of the models by means of suitable tools to trust the DL result. This work presents significant contributions to the field, which are as follows:

- A novel trustworthy DL framework is proposed to detect abnormalities in shoulder X-ray images.
- A new double-in-domain TL approach to overcome previous TL methods’ drawbacks and address the data scarcity issue.
- Consideration of seven pre-trained ImageNet models to be tested with the new TL approach. Furthermore, four different training scenarios are used with all the adopted models.
- The process involves utilising a feature fusion technique that combines features extracted by seven deep neural models. These features are then used to train multiple ML classifiers in four distinct training scenarios.
- The proposed method has shown better results than state-of-the-art methods using the MURA dataset.
- A comprehensive review of state-of-the-art methods for DL in the MURA dataset.
- Three visualisation tools were adopted to validate the robustness of the proposed approach. Grad CAM, LIME, and activation visualisation were used to identify the areas of an image the model utilised for classification. These visualisation tools proved the robustness of the proposed TL.
- Three orthopaedic surgeons were invited to classify the test set and compare their results with the proposed approach. Furthermore, the proposed results were compared with those provided by three radiologists [4], demonstrating a significant improvement over the latter.

## Related work

This section briefly overviews the state-of-the-art methods in the field addressed in our work. Table 1 presents a summary of the revised methods.

**Table 1:** The state-of-the-art methods of the MURA dataset.

Reference	Task	CNN Model	Training	Best results	Limitations
Rajpurkar et al. [4]	Seven MURA tasks	DenseNet-69	Model pre-trained on ImageNet, The MURA dataset was split into 90.7% training, 7.9% testing, and 1.4% validation sets	AU-ROC of 0.92, 0.81 sensitivity and 0.88 specificity	TL from ImageNet.
Kandel et al. [31]	Seven MURA tasks	Six CNN architecture (VGG, Xception, ResNet, GoogLeNet, Inception ResNet, DenseNet)	Two sets of experiments were performed; the first experiment aimed to assess the performance of CNN combinations in averaging, weighted average, or majority vote; the second experiment consisted of using a stacking ensemble.	Accuracy of 84.8% for the elbow dataset using the weighted average votes.	TL from ImageNet, training from scratch, and no performance explainability.
Kandel et al. [32]	Seven MURA tasks	Five state-of-the-art CNNs: VGG19, InceptionV3, ResNet50, Xception, and DenseNet	Training the six architectures from scratch vs the same CNNs pre-trained using the ImagNet.	Xception using TL without FC achieved 83. 5% precision for the elbow images.	TL from ImageNet, training from scratch, and no performance explainability.
He et al. [43]	Seven MURA tasks	ConvNet, ResNet, and DenseNet	Training the three baseline architectures and the proposed calibrated ensemble approach.	AUC: 0.97, accuracy: 0.93, precision: 0.90, recall: 0.97, Cohen’s kappa: 0.85 obtained using the proposed calibrated ensemble model on the humerus dataset.	Not tested against different changes.
Uysal et al. [30]	Shoulder abnormalities	Thirteen DL-based models: ResNet (34,50,101,152), ResNeXt (50,101), DenseNet (169,201), VGG (13,16,19), InceptionV3, and MobileNet V2	Training the 13 CNNs and their fully connected spinal versions and two ensemble models.	Ensemble model EL2: Accuracy: 0.84, Precision: 0.85, Recall: 0.84, F1 score: 0.84, Cohen’s kappa: 0.69.	TL from ImageNet, and no performance explainability.
Malik et al. [44]	Elbow abnormalities	Xception and DarkNetwork-53	Using handcrafted and deep feature fusion and selection based on the whale optimisation approach.	Accuracy of 97.1% and a kappa score of 94.3%	No performance explainability.

Rajpurkar et al. [4] used DenseNet-169 pre-trained on ImageNet and then trained it on a subset of the MURA dataset to predict the probability of abnormality with a prediction probability greater than 0.5 considered abnormal. This model achieved an Area Under the overall Receiver Operator Characteristics (AUROC) of 0.929 with a sensitivity of 0.815 and a specificity of 0.887. In general, the performance of this model was comparable to the performance of radiologists.

Next, numerous studies have used this dataset for different musculoskeletal abnormality detection tasks using different CNN models and TL setups (see Table 1).

In 2019, Varma et al. [42] proposed to use the MURA dataset along with a private dataset of 93455 lower extremity radiographs that includes foot, ankle, knee, and hip data for the detection of abnormality of lower extremity radiographs. The authors tested the model performance of three different CNNs architectures, including ResNet-50, DenseNet-161, and ResNet-101, pre-trained on Imagenet and then trained on a subset of their private dataset. Despite the structural model differences, they found no statistical differences between the performance of these three CNNs architectures. Then, to investigate the effect of TL on model performance, they proposed to compare the performance of DenseNet-161 architecture when pre-trained only on ImagentNet and when pre-trained on ImageNet and then on the MURA dataset. From this experiment, they found that there is no statistically significant difference in model performance when pre-trained on the MURA dataset (for DenseNet pre-trained on ImageNet, values of 0.881, 0.667, and 0.974 were achieved for AUC-ROC, sensitivity, and specificity, respectively, while for DensetNet pre-trained on Imagenet and MURA dataset, values of 0.88, 0.71, and 0.96 were achieved for AUC-ROC, sensitivity, and specificity, respectively). Furthermore, to investigate the effect of size on the performance of the pre-trained model using MURA, DenseNet-161 was trained in subsets of the lower extremity dataset of different sizes (1,000, 5,000, 10,000, and 50,000). The results showed that for small training datasets, pretraining in MURA significantly increased performance (AUC-ROC risen from 0.67 to 0.78 for the subset of 1000). The study findings suggested that TL presents a promising strategy for improving the model performance for the abnormality detection task when the labelled training dataset is limited.

To highlight the importance of TL in classifying X-ray images, Kandel et al. [31] used the MURA dataset to investigate the performance of six CNN architectures ((i.e. VGG, Xception, ResNet, GoogLeNet, InceptionResNet, and DenseNet) to detect bone abnormalities with models trained from scratch against the same CNN architectures pre-trained using ImageNet. Furthermore, for each test dataset, two experiments were performed. After each architecture, the first experiment added a fully connected (FC) layer, and the second involved adding a sigmoid function. The overall best accuracy of 83.5% was achieved using a fine-tuned Xception architecture without an FC layer for the elbow images. For classifying shoulder images, the best result in terms of accuracy was 79.2% and was achieved using a fine-tuned DenseNet architecture with an FC layer. In the study, it was shown that TL is capable of increasing model performance while making it less prone to overfitting.

Kandel et al. [32] investigated the performance of five state-of-the-art CNNs ((i.e. VGG19, InceptionV3, ResNet50, Xception, and DenseNet) for the same classification task. Two sets of experiments were performed. The first experiment aimed to assess the combination of CNN performance by averaging, weighted averaging, or using a majority vote. The second experiment consists of using a stacking ensemble. The classification was performed for each of the MSK image categories of the MURA dataset. The best-obtained precision was 84.8% for the elbow dataset using weighted average votes. For the shoulder classification task, the GBM classifier achieved the best accuracy of 75.2%. This study proposed using different ensemble techniques to improve the classification of musculoskeletal abnormalities rather than relying on a single CNN classification.

He et al. [43] used three CNN architectures (i.e. ConvNet, ResNet and DenseNet) and proposed a calibrated ensemble approach for detecting musculoskeletal abnormalities. The authors found that the proposed model outperformed the three state-of-the-art architectures with outperform performance in the humerus dataset (AUC: 0.97, Accuracy: 0.93, Precision: 0.90, Recall: 0.97, Cohen’s kappa: 0.85). Similarly, an AUC of 0.90, an accuracy of 0. 85% and a precision of 0.86 were obtained using ResNet and the proposed model that deals with the shoulder classification task.

A more focused study on the shoulder joint by Uysal et al. [30] investigated TL using the MURA dataset to detect shoulder abnormalities. This study aims to examine the DL ensemble models for the shoulder X-ray classification task. A total of 26 DL-based models (ResNet-34,50,101,152, ResNeXt-50,101, DenseNet-169,201, VGG-13,16,19, InceptionV3, MobileNetV2, and their fully connected spinal (Spinal-FC versions) were used, and their performances were evaluated. Thus, two ensemble models were proposed using the pre-trained models with the best performance. Only X-ray images of the shoulder bone were utilised from the MURA dataset and were divided into training and testing. For baseline models with standard FC and Spinal-FC, the highest results were achieved using DenseNet169 (Accuracy: 0.84, Precision: 0.84, Recall: 0.84, F1 score: 0.84, Cohen’s kappa: 0.68) among models with standard FC and using DenseNet201 (Accuracy: 0.82, Precision: 0.83, Recall: 0.83, F1 score: 0.83, Cohen’s kappa: 0.65) among models with Spinal FC. Both proposed ensemble models outperformed the baseline models, with the second model, EL2, achieving the highest performance (accuracy: 0.84, precision: 0.85, recall: 0.845, F1 score: 0.84, Cohen’s kappa: 0.69). The promising results of the shoulder task achieved explicitly in these studies encourage the use of TL to detect shoulder abnormalities. However, the pre-processing step used to eliminate the noise and dark background limits the usage of this model in a more generic context and makes the comparison with other studies in the literature misleading due to the difference in the test dataset.

Recently, Malik et al. [44] used a subset of 16984 elbow X-ray radiographs from the MURA dataset to test the model’s performance to classify elbow abnormalities. First, they proposed adding a pre-processing step to convert images to RGB colour space. Then, the Xception and DarkNetwork-53 architectures were used to extract deep features. Similarly, two hand-crafted features, including texture and shape-based features, were extracted from the input images, and principal component analysis was used for the best feature selection. These features were serially merged, and then feature selection was performed using the whale optimisation approach (WOA) and supplied to support vector machine, K-nearest neighbour, and wide neural network (WNN) classifiers. The performance of the proposed method was evaluated on X-ray radiographs of the elbow. The model obtained an accuracy of 97.1% with a kappa score of 94.3%.

Manoila, C. et al. [45] introduced a flexible MRI analysis framework for automated delineation of the knee joint region, featuring various DL models with preset parameters. It highlights a promising convolutional neural network (CNN) for knee bone segmentation and a novel weighted downsampling method to improve image processing.

## Motivation

Detecting shoulder abnormalities through X-ray images can be both a challenging and a time-consuming task. Radiologists and physicians use radiographs in orthopaedic routines to assess bone anatomy and detect joint abnormalities. Assessing the imaging becomes challenging, especially in an emergency setting with a high volume of care overload. Subsequently, the experience level in interpreting these images could vary, and accuracy could be affected.

On the other hand, DL has demonstrated outstanding performance in several tasks, including the application of X-ray images, which are also used to detect shoulder fractures. DL requires a large amount of data to perform well, which is the reason for the poor performance of previous methods in detecting shoulder abnormalities (such as fractures).

Furthermore, TL from the ImageNet dataset was used to solve the issue, which was proved ineffective due to the mismatch between the colour features of ImageNet and greyscale X-ray images.

Lastly, DL models are often called “black boxes” because the reasoning behind their decision-making is not always transparent. Establishing trust in these models before deploying them is crucial by providing clear evidence on how decisions are made. However, most previous methods have failed to explain how models make decisions clearly. Therefore, these reasons motivated us to address these pitfalls and drawbacks to improve the performance of the detection of shoulder abnormalities.

## Materials and methods

### Dataset

MURA is a large dataset of bone X-rays [4]. The MURA dataset contains seven skeletal bones: elbow, finger, forearm, hand, humerus, shoulder, and wrist. Each part has been divided into two subclasses: positive and negative. The total number of images is 40,561. The dataset was split into training and test sets, as explained in Table 2.

**Table 2:** Number of images of the MURA dataset.

Type of Bone	Training		Testing	
	Negative	Positive	Negative	Positive
Elbow	2925	2006	234	230
Finger	3138	1968	214	247
Hand	4059	1484	271	189
Humerus	673	599	148	140
Forearm	1164	661	150	151
Shoulder	4211	4168	285	278
Wrist	5765	3987	364	295

**Fig 1:**
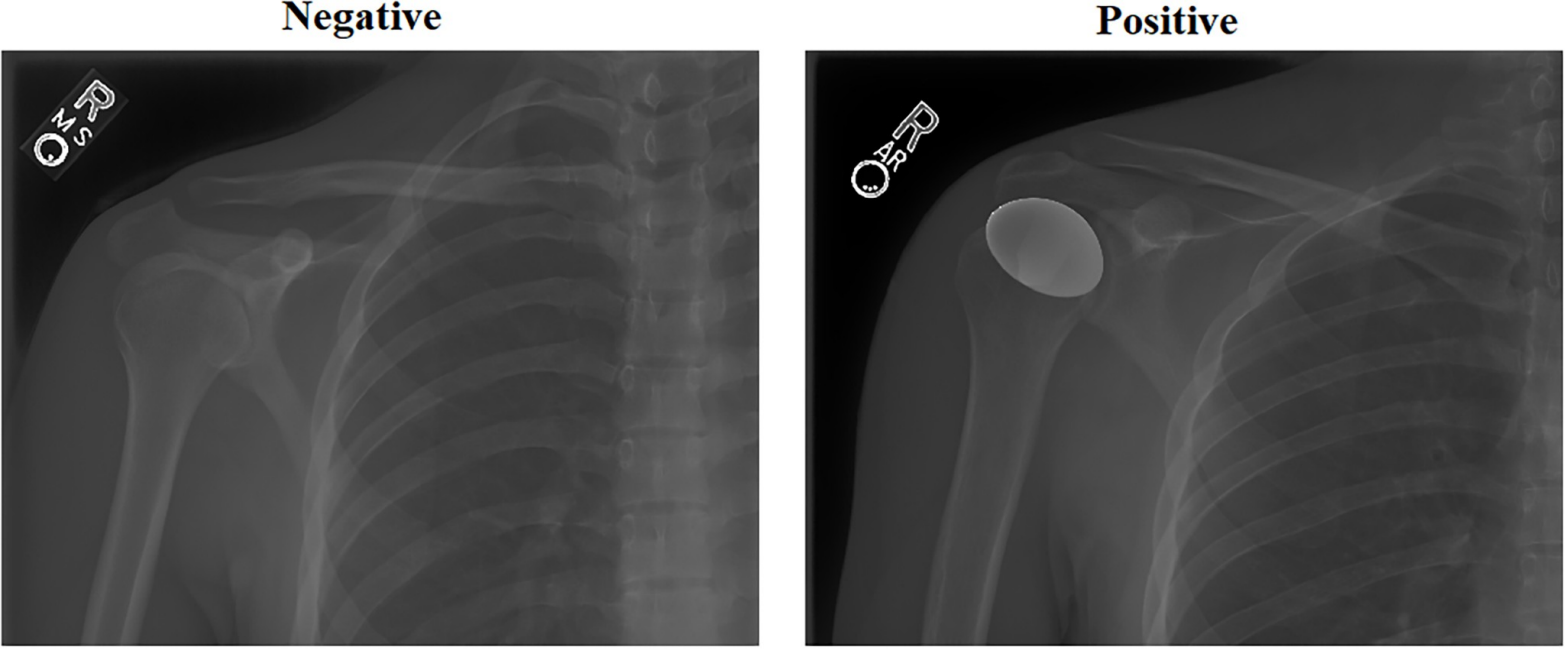
Two samples of the shoulder category.

The dataset was divided into two major groups as follows:

- Target dataset: The shoulder category has been considered a target dataset. This is because the shoulder category is the most balanced. Two samples from the shoulder category are shown in Fig 1. We have used the same dataset division as the initial setup and have implemented a portion of the training set as a validation set.
- Source of TL: All other categories have been considered for the source of TL. This step will help to update the features of pre-trained models of ImageNet to be relevant to the target dataset.

### The proposed DL framework

**Fig 2:**
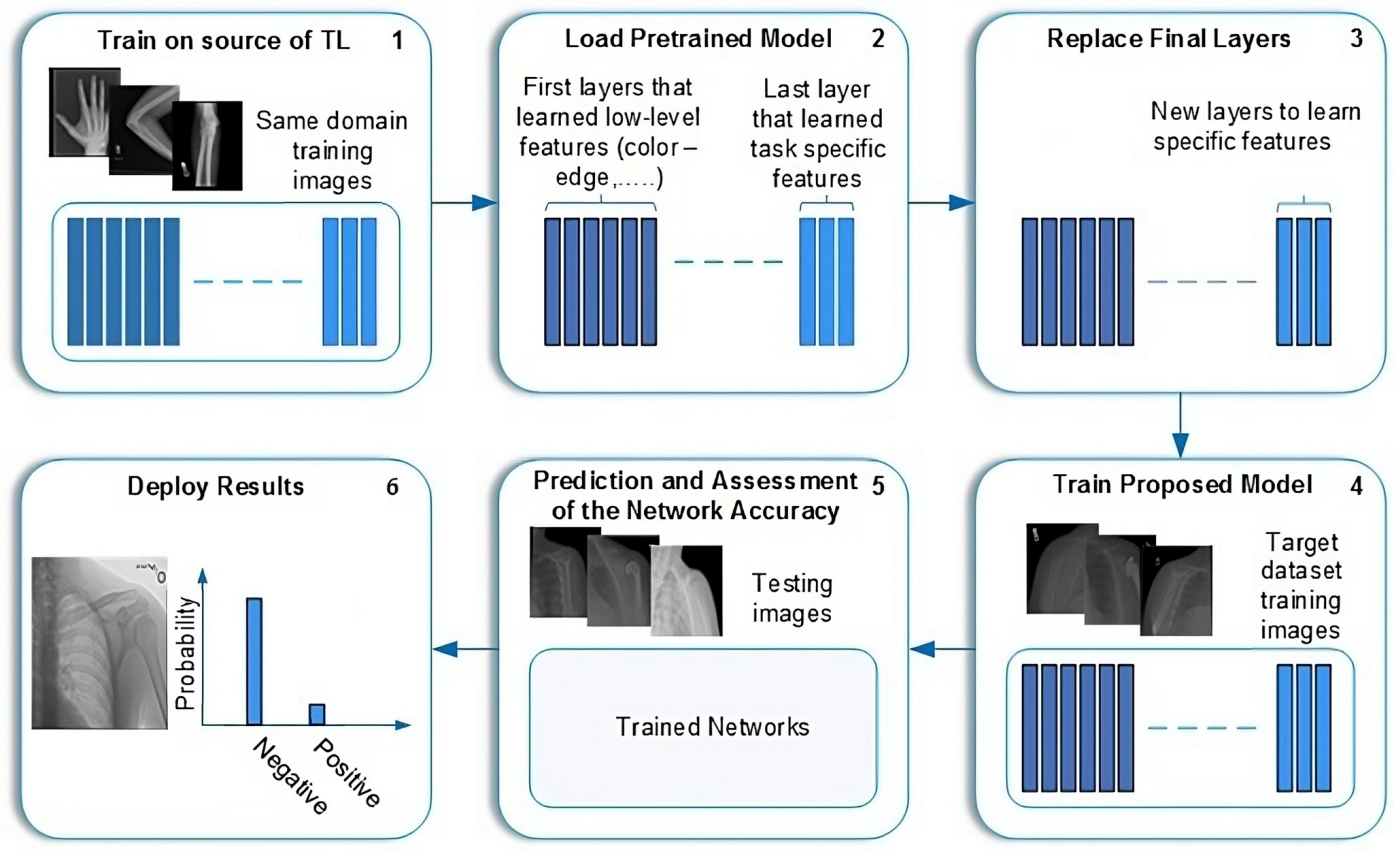
Fine-tuning process.

**Fig 3:**
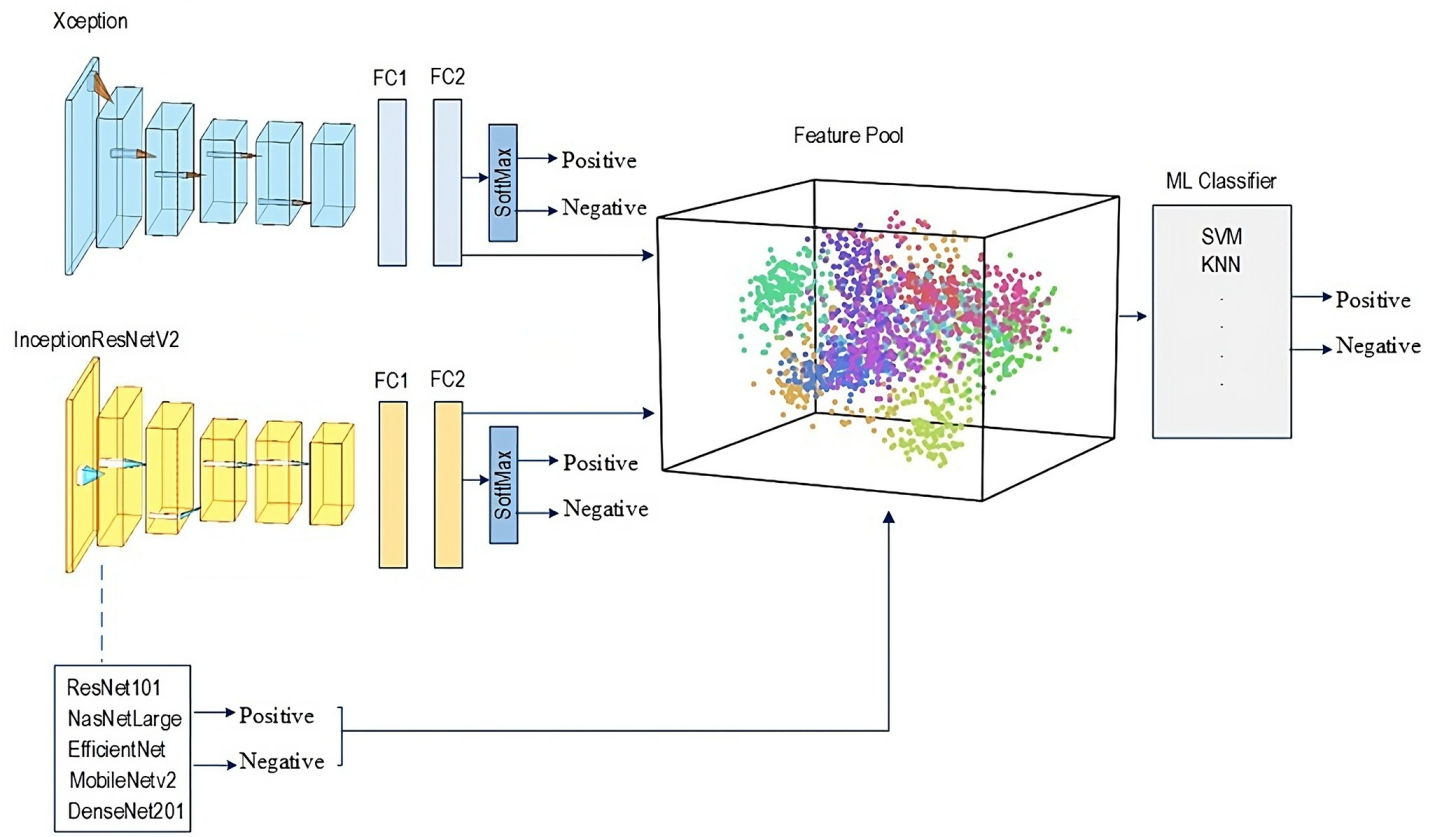
Feature fusion process.

The DL framework proposal consists of two parts, which are explained below:

- Part 1: Proposed TL
  TL is learning from a large dataset and then transferring the knowledge to a small dataset. One of the most common demonstrations of TL is the pre-trained models of the ImageNet dataset. It consists of 1,000 classes of natural images, including various humans, plants, animals, etc., with millions of images. Several successful tasks, such as in agriculture and object detection, utilised the pre-trained models of the ImageNet dataset to tackle the issue of data scarcity. TL from the pre-trained models of ImageNet cannot be helpful if the target task dataset does not have relevant features with ImageNet. For instance, the ImageNet dataset is a colour dataset that cannot improve the performance of greyscale medical images, such as X-ray, CT, MRI, etc. There is an apparent mismatch between the learning features.
  This paper proposes a new adaptation of the TL domain to overcome this challenge and improve the results. The proposal is based on updating the features of the pre-trained models of ImageNet using in-domain images and then fine-tuning the models (see Fig 2) for the target dataset. All tasks in the MURA dataset, except the shoulder, have been used as a source of TL. The models will then be fine-tuned and trained on the target dataset of shoulder tasks. Our method guarantees that the models learn relevant features, as both the source of TL and the target task use the same image modality (X-ray) and share the same aim of abnormality detection. It also reduces the need for annotated images of the target task. The source of TL can be time-efficient unannotated images.
  Seven pre-trained models trained with and without the proposed TL were employed. These models have been chosen based on their performance using the ImageNet dataset. Our study considers various sizes, depths, and image input sizes, as explained in Table 3. The primary motivation is to test the proposed TL with different models.
- Part 2: Proposed Deep-Feature Fusion
  ML-based classification techniques require fully descriptive features to distinguish between classes to achieve high performance. To accomplish this, the feature fusion technique is used to enhance the results of individual models. This technique enables a complete description of the internal information, resulting in a compact representation of fused features, thus improving the performance of this task. Seven deep convolutional neural networks have been individually trained and evaluated. The trained models are used to extract features. The features extracted from the seven models have been fused into one group to train ML classifiers. Several ML classifiers have been adopted, including Decision Tree, Linear Discriminant, Naive Bayes, SVMs, K-Nearest Neighbour, Logistic Regression, and Neural Networks. Fig 3 depicts the fusion process.
  The feature fusion technique offers several advantages. First, it allows flexibility in incorporating additional DL models into the system, which can expand and enhance the feature representation. This means that as new DL models become available or the dataset grows, they can be integrated into the system, improving overall performance and accuracy. Second, it enhances the representation of features by combining the unique and complementary information captured by each DL model. This leads to a more comprehensive and discriminative representation of the image. Additionally, it eliminates the need to train models from scratch when incorporating additional data. This significantly reduces training time and computational resources, making the system more efficient and scalable.

**Table 3:** Details of the pre-trained models of ImageNet.

Model	Input Size	Parameters (10^6^)	Depth
Xception	299 × 299 × 3	22.9	71
Inception-ResNetV2	299 × 299 × 3	55.9	164
MobilNetV2	224 × 224 × 3	3.5	53
EfficientNetb0	224 × 224 × 3	5.3	82
DenseNet201	224 × 224 × 3	20.0	201
ResNet101	224 × 224 × 3	44.6	101
NasNetlarge	331 × 331 × 3	88.9	1244

### Training scenarios

**Fig 4:**
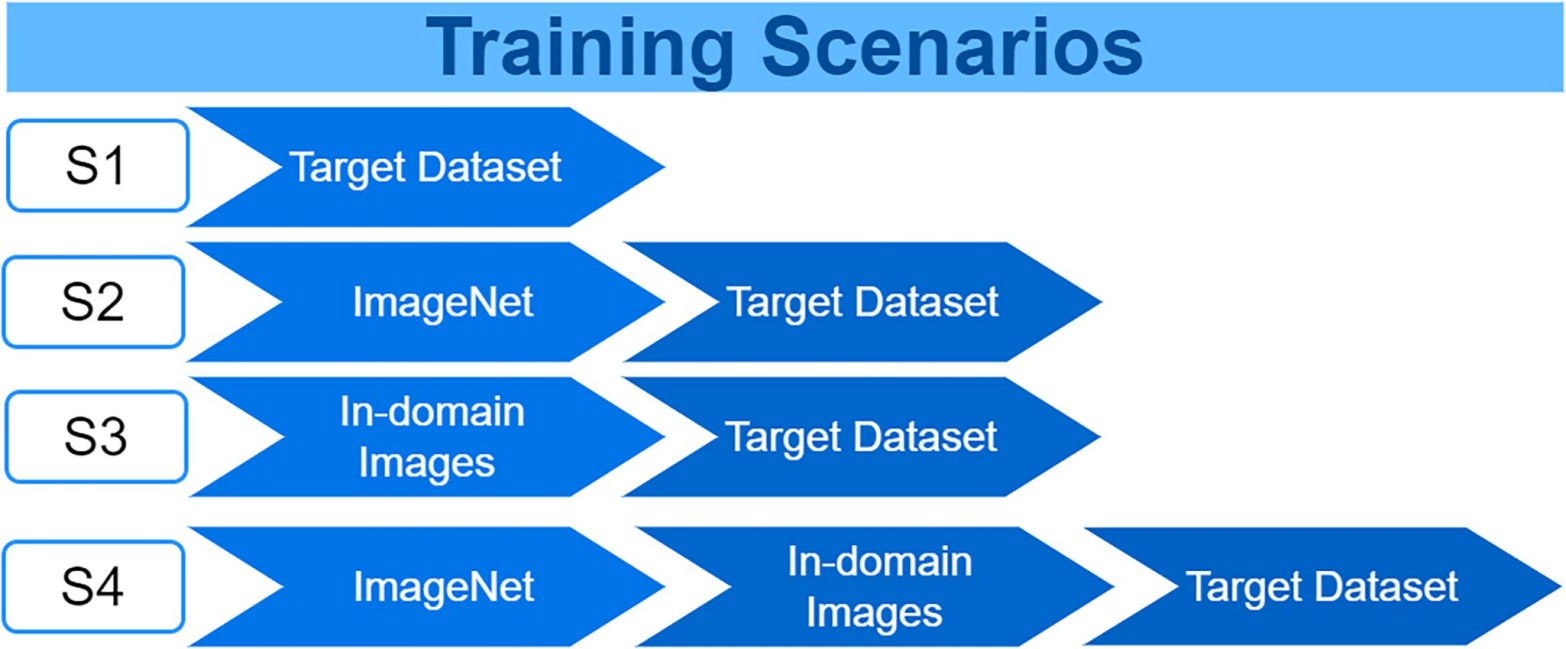
The four different training scenarios.

The dataset employed in this study has been divided into three distinct sets: training, validation, and testing. This paper explores four distinct training scenarios, each contributing to a comprehensive understanding of the models’ performance. These scenarios are visualised in Fig 4:

- **Scenario 1 (S1)**: Training the ImageNet models from scratch on the target dataset.
- **Scenario 2 (S2)**: Training of ImageNet models using TL from the ImageNet dataset on the target dataset.
- **Scenario 3 (S3)**: Training the ImageNet models from scratch with TL source collection (in-domain images) and then training on the target dataset.
- **Scenario 4 (S4)**: Training the ImageNet models using TL from the ImageNet dataset to train with TL source collection (in-domain images), then training on the target dataset.

**Fig 5:**
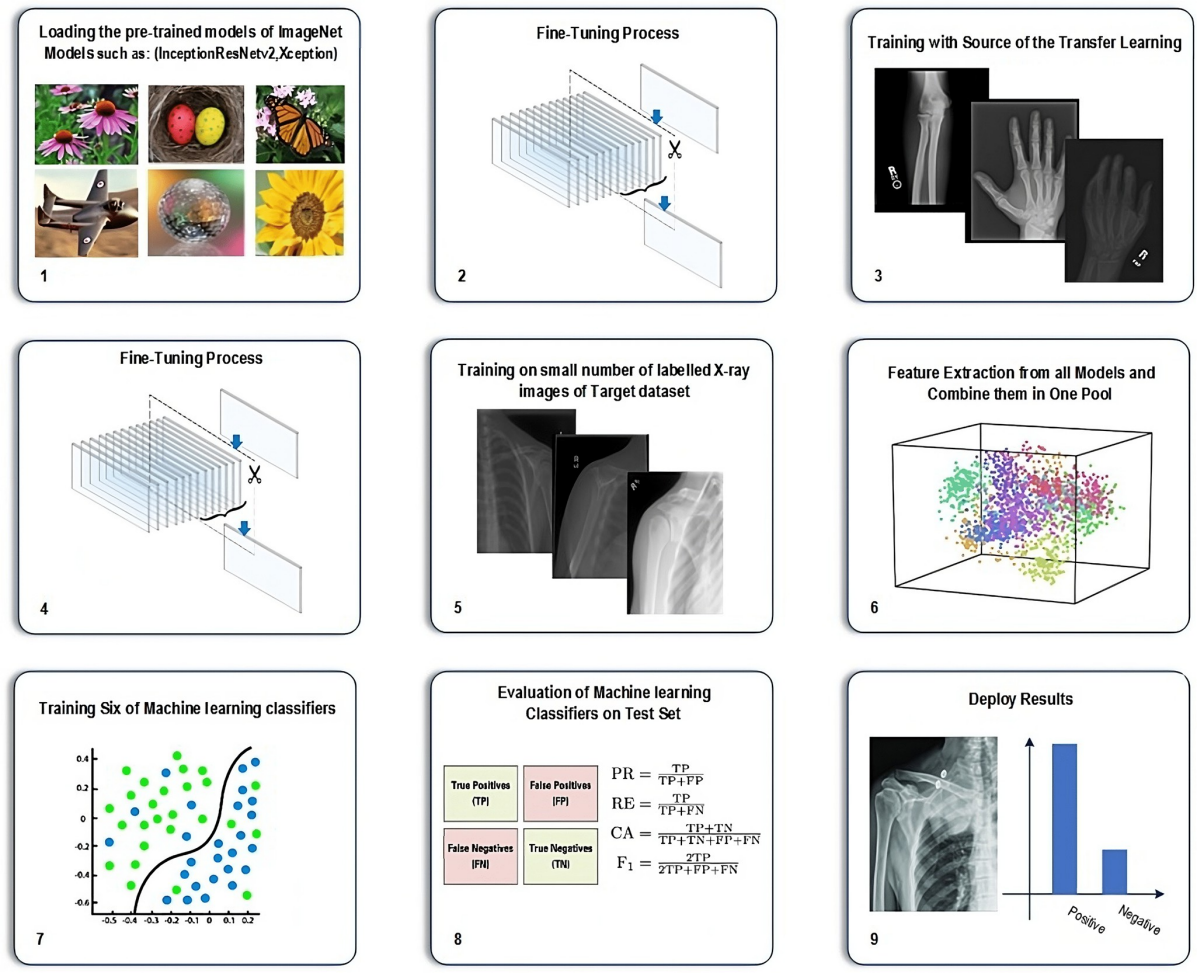
Workflow of Scenario 4 (S4) using feature fusion.

The training hyperparameters are Adam optimiser, mini-batch size of 15, max epochs of 100, Shuffle for every epoch, and the initial learning rate of 0.001. Fig 5 shows scenario 4 (S4) workflow with feature fusion. The processor properties used in this experiment are Intel (R) Core i7/32GB/1TB/Nvidia RTX A3000 12GB. Matlab 2022a was used to run the experiments.

### Visualisation techniques for explainable deep learning models

**Fig 6:**
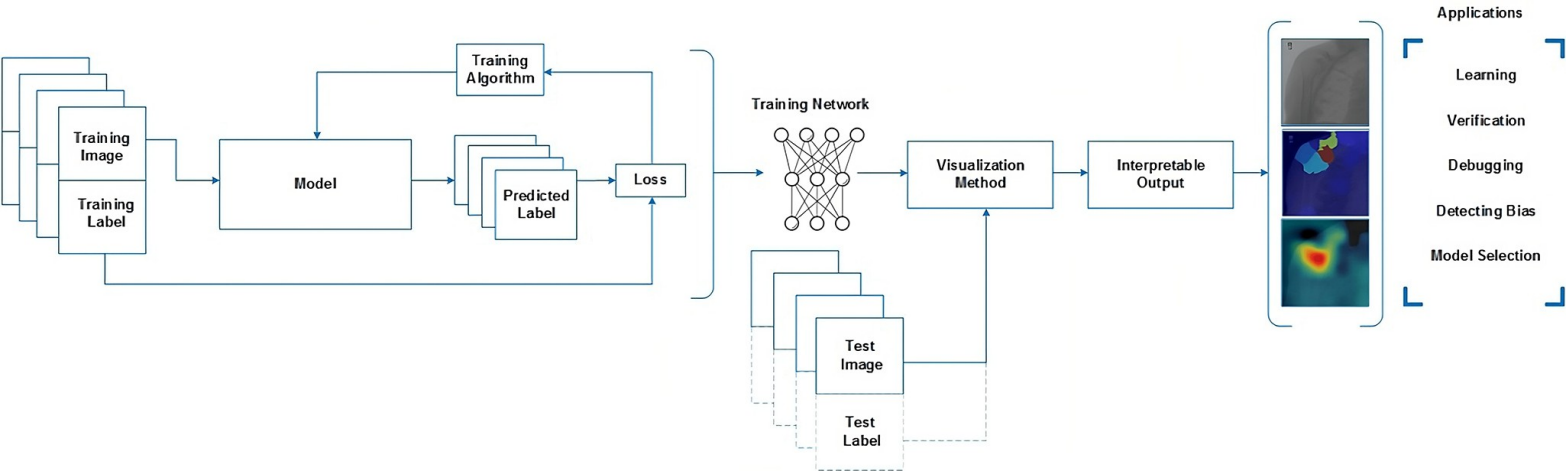
Workflow of visualisation techniques.

DL models are like “black boxes” because their decision-making process is often unclear. Trusting DL models before deploying them beyond the research phase is essential. Post-training methods that use test images can be utilised for verification, debugging, learning, bias assessment, and model selection. This article focuses on post-training methods that use test images to explain the predictions of a network trained on image data, as shown in Fig 6. Three different visualisation techniques are used to validate whether the models are looking at the region of interest to make the decision as listed below:

1. Grad-CAM uses the gradient of the classification score about the convolutional features specified by the model to realise which regions of the image are most important for making the decision. The regions where the gradient is large are the places where the final score largely depends on the data.
2. Activation visualisation is a straightforward technique to understand the model’s behaviour. The first convolutional layer usually learns simple features like colour and edges, while the last one learns more complex features.
3. The LIME technique approximates the classification behaviour of a DL model using a simpler, more interpretable model, such as a linear model or a regression tree. The simple model defines the significance of the features of the input image as a proxy for the significance of the features to the DL model.

## Experimental assessment

This section is devoted to the experimental evaluation of the proposed TL approach in detecting abnormalities in the shoulder.

### Evaluation metrics

All models with different training scenarios were evaluated based on precision, specificity, recall, precision, and F1 score metrics. These evaluation metrics were calculated based on the TN, TP, FN, and FP values. TN and TP implied precisely categorised negative and positive instances, while FN and FP denoted misclassified positive and negative cases, respectively. Every evaluation metric equation is presented as follows:

$$\begin{array}{c} Accuracy=\frac{TP+TN}{TP+TN+FP+FN} \end{array}$$ (1)

$$\begin{array}{c} Specificity=\frac{TN}{FP+TN} \end{array}$$ (2)

$$\begin{array}{c} Recall=\frac{TP}{TP+FN} \end{array}$$ (3)

$$\begin{array}{c} Precision=\frac{TP}{TP+FP} \end{array}$$ (4)

$$\begin{array}{c} F1_{score}=2\times \frac{Precision\times Recall}{Precision+Recall} \end{array}$$ (5)

Cohen’s kappa equation:

$$\begin{array}{c} K_{0}=\frac{TP+TN}{TP+TN+FP+FN} \end{array}$$ (6)

$$\begin{array}{c} K_{positive}=\frac{\left(TP+FP)(TP+FN\right)}{{\left(TP+TN+FP+FN\right)}^{2}} \end{array}$$ (7)

$$\begin{array}{c} K_{negative}=\frac{\left(FN+TN)(FP+TN\right)}{{\left(TP+TN+FP+FN\right)}^{2}} \end{array}$$ (8)

$$\begin{array}{c} K_{e}=K_{positive}+K_{negative} \end{array}$$ (9)

Cohen’s kappa score=

$$\begin{array}{c} \frac{K_{0}-K_{e}}{1-K_{e}} \end{array}$$ (10)

### Part 1: Experimental assessment of end-to-end DL models

Seven DL models (see Table 3) have been evaluated with four training scenarios as described in Table 4.

**Fig 7:**
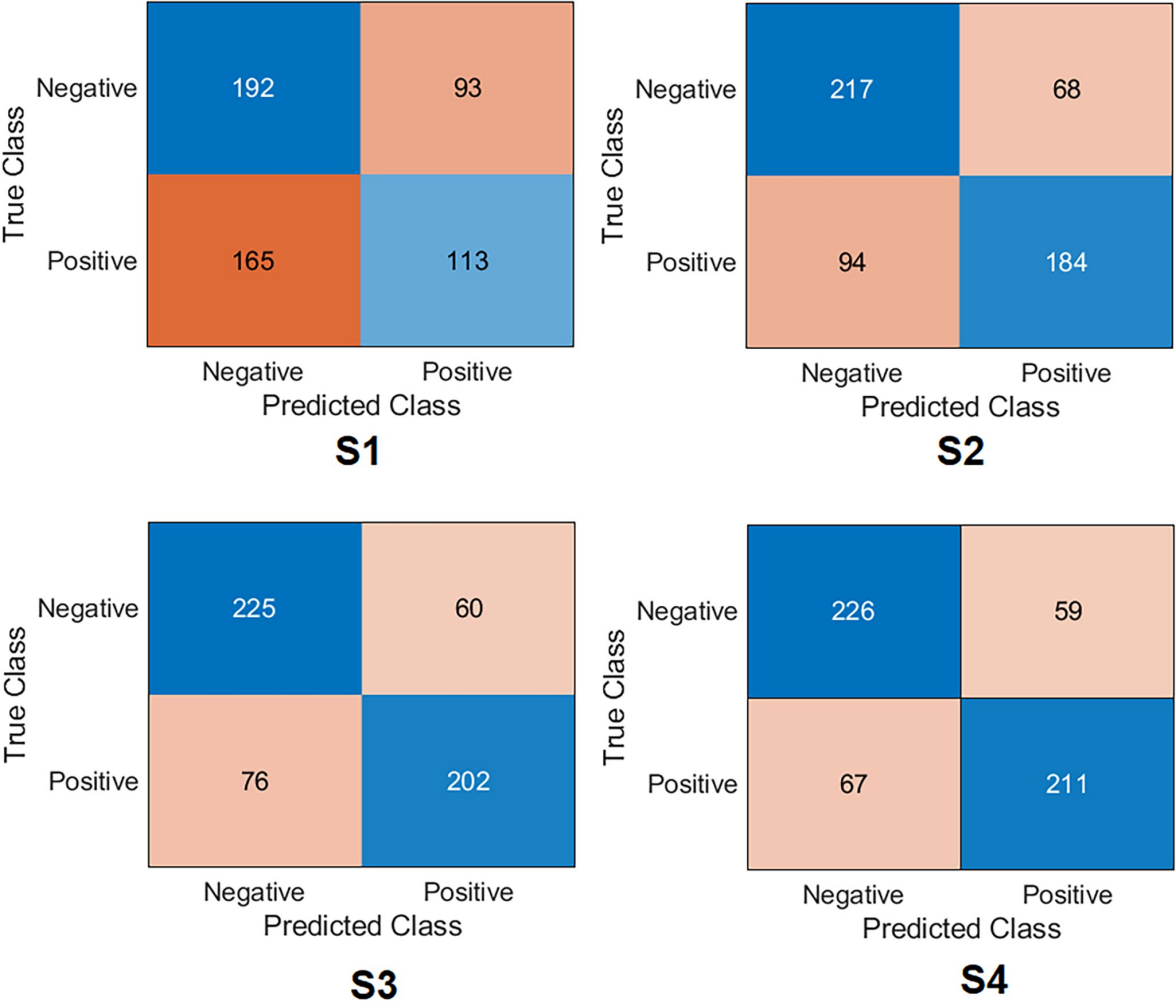
Confusion matrix of the Xception model on the test set with four training scenarios.

**Fig 8:**
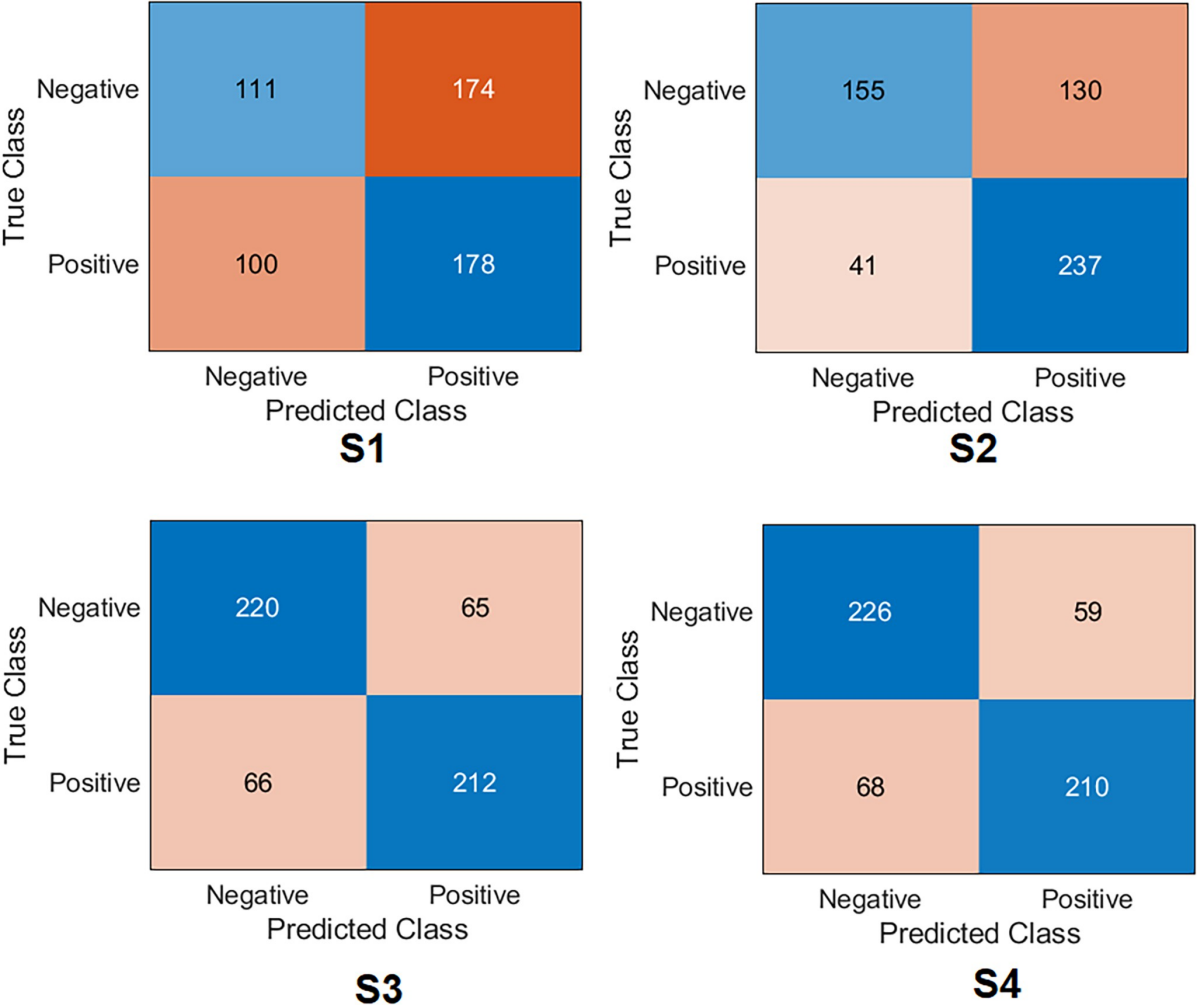
Confusion matrix of the InceptionResNetV2 model on the test set with four training scenarios.

**Fig 9:**
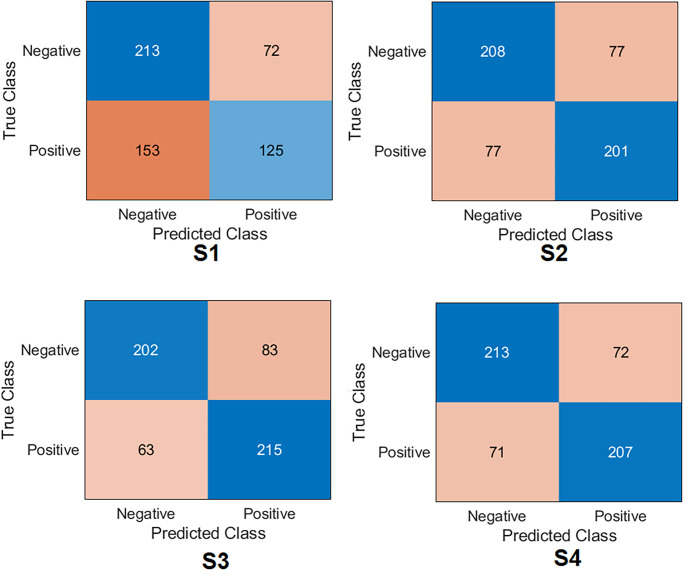
Confusion matrix of the MobilNetV2 model on the test set with four training scenarios.

**Fig 10:**
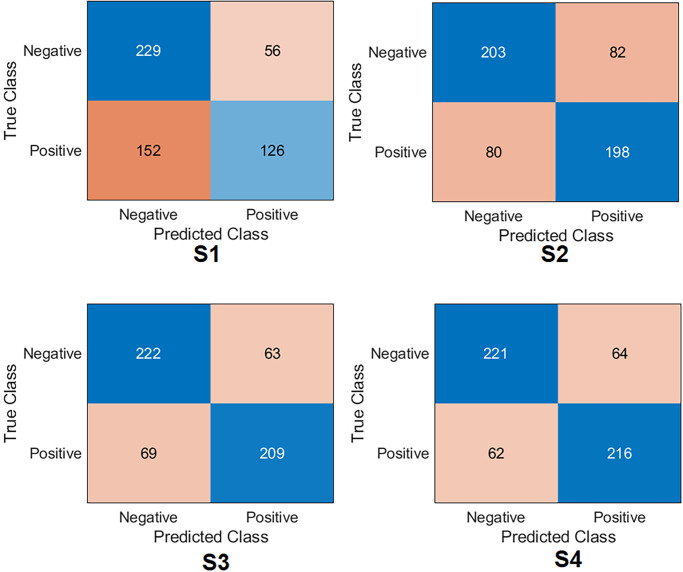
Confusion matrix of the EfficientNet model on the test set with four training scenarios.

**Fig 11:**
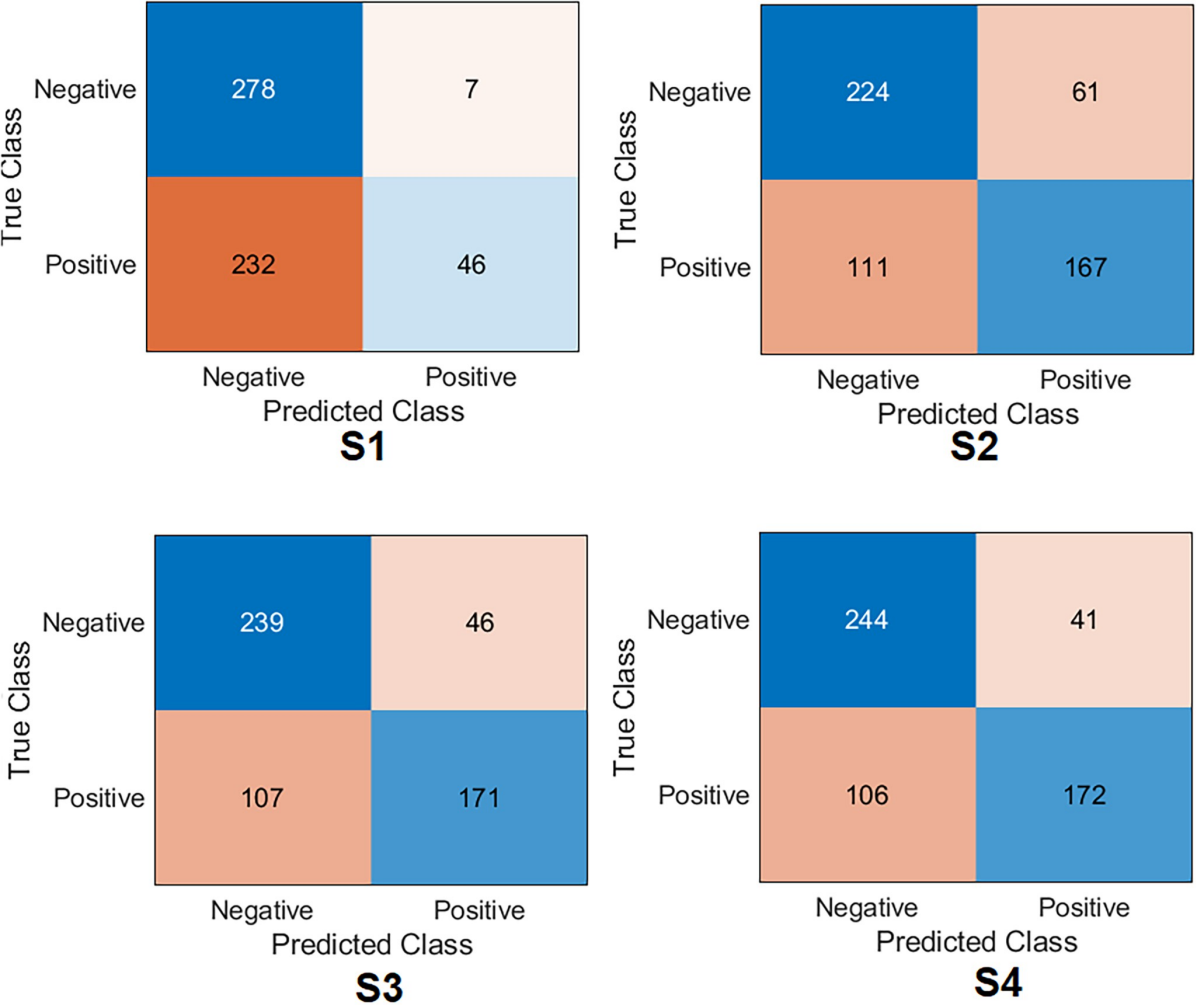
Confusion matrix of the DenseNet201 model on the test set with four training scenarios.

**Fig 12:**
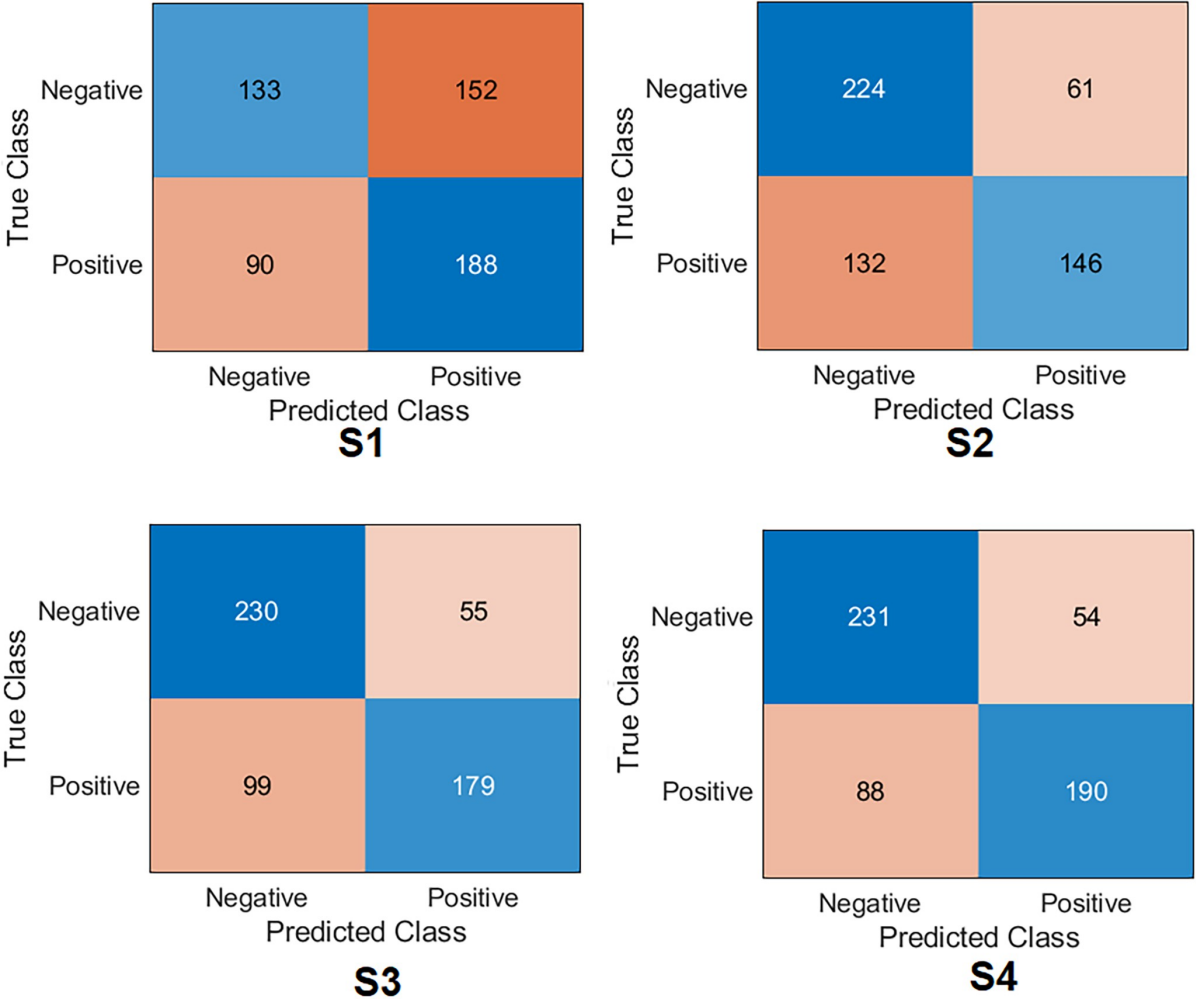
Confusion matrix of the ResNet101 model on the test set with four training scenarios.

**Fig 13:**
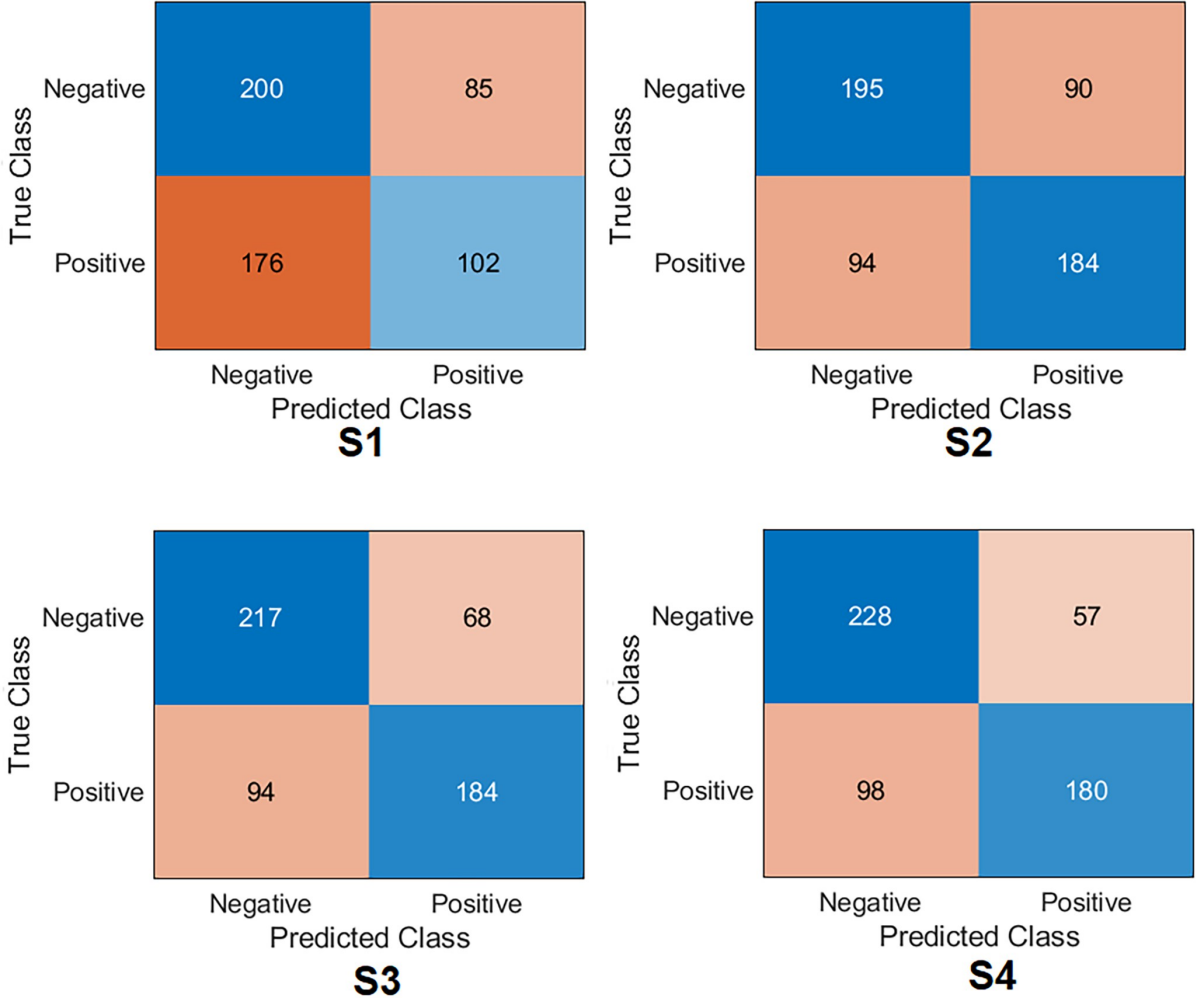
Confusion matrix of the NasNetLarge model on the test set with four training scenarios.

1. **Xception Case:** The confusion matrix was first calculated for all training scenarios as shown in Fig 7. Based on the values of the confusion matrix, the evaluation metrics have been calculated, showing that S4 achieved the highest results, obtaining an accuracy of 77.6%, specificity of 79.3%, recall of 75.9%, precision of 78.1%, *F*1_*score*_ of 77.0% and Cohen’s kappa of 55.2%. S3 obtained 75.8%, 78.9%, 72.6%, 77.1%, 74.8%, and 51.6% for accuracy, specificity, recall, precision, *F*1_*score*_ and Cohen’s kappa, respectively. These results put S3 second after S4. S2 achieved third place by obtaining an accuracy of 71. 2%, a specificity of 76.1%, a recall of 66.1%, a precision of 73.0%, *F*1_*score*_ of 69.4%, and Cohen’s kappa of 42.3%. S1 achieved the lowest results compared to the other scenarios by obtaining an accuracy 54. 2%, a specificity of 67.3%, a recall of 40.6%, a precision of 54.8%, *F*1_*score*_ of 46.6%, and Cohen’s kappa of 8.04%.
2. **InceptionResNetV2 Case:** The confusion matrix was first calculated for all training scenarios, as shown in Fig 8. Similarly to the Xception model, S4 also achieved the highest results by obtaining an accuracy of 77.4%, a specificity of 79.3%, and a recall of 75. 5%, a precision of 78. 1% and *F*1_*score*_ of 76.8%. S3 obtained 76. 7%, 77.1%, 76.2%, 76.5%, 76. 4% for precision, specificity, recall, precision, *F*1_*score*_, respectively. These results keep S3 in second place after S4. S2 achieved third place by obtaining an accuracy of 69. 6%, a specificity of 54. 3%, a recall of 85. 2%, a precision of 64. 5% and *F*1_*score*_ of 73.4%. S1 also achieved the lowest results compared to other scenarios by obtaining an accuracy of 51.3%, a specificity of 38. 9%, a recall of 64.0%, a precision of 50. 5% and *F*1_*score*_ of 56.5%. Regarding Cohen’s kappa, S4 obtained the highest value by achieving 54.8%; S3 achieved 53.4%, S2 achieved 39.4%, and S1 achieved 2.96%.
3. **MobilNetV2 Case:** The confusion matrix was first calculated for all training scenarios, as shown in Fig 9. With the same flow as in the previous models, S4 was kept as the top scenario, achieving an accuracy of 74.6%, specificity of 74.7%, recall of 74.4%, with a precision of 74.2% and *F*1_*score*_ of 74.3%. S3 was second on the list, achieving 74.1%, 70.8%, 77.3%, 72.1%, and 74.6% for precision, specificity, recall, precision, and *F*1_*score*_. S2 achieved third place by obtaining an accuracy of 72.6%, specificity of 72.9%, and recall of 72. 3%, the precision of 72.3% and *F*1_*score*_ of 72.3%. S1 maintained the lowest results compared to other scenarios by obtaining an accuracy of 60.0%, a specificity of 74.7%, a recall of 44.9%, with a precision of 63.4% and *F*1_*score*_ of 52.6%. Regarding Cohen’s kappa, S4 obtained the highest value by achieving 49.1%; S3 achieved 48.1%, S2 achieved 45.2%, and S1 achieved 19.7%.
4. **EfficientNet Case:** The confusion matrix was first calculated for all training scenarios, as shown in Fig 10. With the same flow as the previous models, S4 was the top scenario, achieving an accuracy of 77.6%, specificity of 77.5%, recall of 77.7%, precision of 77.1%, and *F*1_*score*_ of 77.4%. S3 was second on the list, achieving 76.5%, 77.8%, 75.1%, 76. 8%, 76. 0% for accuracy, specificity, recall, precision, and *F*1_*score*_. S2 achieved third place by obtaining an accuracy of 71.2%, a specificity of 71.2%, a recall of 71.2%, a precision of 70.7%, and *F*1_*score*_ of 70.9%. Again, S1 had the lowest results compared to other scenarios, obtaining an accuracy 63. 0%, a specificity of 80.3%, a recall of 45.3%, a precision of 69.2%, and *F*1_*score*_ of 54.7%. In terms of Cohen’s kappa, S4 obtained the highest value by achieving 55.2%; S3 achieved 53.08%, S2 achieved 42.4%, and S1 achieved 25.7%.
5. **DenseNet201 Case:** The confusion matrix was first calculated for all training scenarios, as shown in Fig 11. Similarly to previous models, S4 also achieved the highest results by obtaining an accuracy of 73.8%, a specificity of 85.6%, a recall of 61.8%, a precision of 80. 7% and *F*1_*score*_ of 70.0%. S3 obtained 72. 8%, 83. 8%, 61. 5%, 78. 8%, and 69.0% for precision, specificity, recall, precision, and *F*1_*score*_. These results keep S3 in second place after S4. S2 achieved third place by obtaining an accuracy of 69.4%, a specificity of 66. 8%, a recall of 78.6%, a precision of 73.2%, and *F*1_*score*_ of 72.2%. S1 achieved the lowest results compared to other scenarios by obtaining a precision of 57.5%, a specificity of 97. 5%, a recall of 16. 5%, a precision of 86.7%, and *F*1_*score*_ of 27.7%. In terms of Cohen’s kappa, S4 obtained the highest value by achieving 47.6%; S3 achieved 45.4%, S2 achieved 38.7%, and S1 achieved 14.2%.
6. **ResNet101 Case:** The confusion matrix was first calculated for all training scenarios, as shown in Fig 12. S4 achieved the highest results by obtaining an accuracy of 74.7%, a specificity of 81. 0%, a recall of 68. 3%, a precision of 77.8%, and *F*1_*score*_ of 72.8%. S3 was second in the list, achieving 72.6%, 80.7%, 64.3%, 76.5% and 69. 9% for accuracy, specificity, recall, precision, and *F*1_*score*_. S2 achieved third place by obtaining an accuracy of 65.7%, a specificity of 78. 6%, a recall of 52.5%, a precision of 70.5%, and *F*1_*score*_ of 60.2%. S1 displayed the lowest results compared to other scenarios by obtaining a precision of 57. 0%, specificity of 46.6%, recall of 67. 6%, the precision of 55.2%, and *F*1_*score*_ of 60.8%. In terms of Cohen’s kappa, S4 obtained the highest value by achieving 49.4%; S3 achieved 45.1%, S2 achieved 31.2%, and S1 achieved 14.2%.
7. **NasNetLarge Case:** The confusion matrix was first calculated for all training scenarios, as shown in Fig 13. S4 also achieved the highest results by obtaining an accuracy of 72.4%, a specificity of 80.0%, a recall of 64.7%, a precision of 75.9% and *F*1_*score*_ of 69.9%. S3 is second in the list, achieving 71.2%, 76.1%, 66.1%, 73.0%, and 69.4% for precision, specificity, recall, precision and *F*1_*score*_. S2 achieved third place by obtaining an accuracy of 67. 3%, a specificity of 68.4%, a recall of 66.1%, a precision of 67.1%, and *F*1_*score*_ of 66.6%. S1 maintained the lowest results compared to other scenarios by obtaining a precision of 53.6%, a specificity of 70.1%, a recall of 36.6%, a precision of 54.5% and *F*1_*score*_ of 43.8%. In terms of Cohen’s kappa, S4 obtained the highest value by achieving 44.8%; S3 achieved 42.3%, S2 achieved 34.6%, and S1 achieved 6.89%.

**Table 4:** Results of DL models in the shoulder task test set of the MURA dataset.

Evaluation Metric (%)	Xception			
-	S1	S2	S3	S4
Accuracy	54.2	71.2	75.8	77.6
Specificity	67.3	76.1	78.9	79.3
Recall	40.6	66.1	72.6	75.9
Precision	54.8	73.0	77.1	78.1
F1 _*Score*_	46.6	69.4	74.8	77.0
Cohen’s kappa	8.04	42.3	51.6	55.2
-	InceptionResNetV2			
Accuracy	51.3	69.6	76.7	77.4
Specificity	38.9	54.3	77.1	79.3
Recall	64.0	85.2	76.2	75.5
Precision	50.5	64.5	76.5	78.1
F1 _*Score*_	56.5	73.4	76.4	76.8
Cohen’s kappa	2.96	39.4	53.4	54.8
-	MobilNetV2			
Accuracy	60.0	72.6	74.1	74.6
Specificity	74.7	72.9	70.8	74.7
Recall	44.9	72.3	77.3	74.4
Precision	63.4	72.3	72.1	74.2
F1 _*Score*_	52.6	72.3	74.6	74.3
Cohen’s kappa	19.7	45.2	48.1	49.1
-	EfficientNetb0			
Accuracy	63.0	71.2	76.5	77.6
Specificity	80.3	71.2	77.8	77.5
Recall	45.3	71.2	75.1	77.7
Precision	69.2	70.7	76.8	77.1
F1 _*Score*_	54.7	70.9	76.0	77.4
Cohen’s kappa	25.7	42.4	53.08	55.2

Several conclusions can be highlighted from Tables 4 and 5:

- Each of the seven models tested achieved high results with S4, demonstrating that the proposed TL has proven the results by learning relevant features.
- Even though the pre-trained models of ImageNet learned features irrelevant to X-ray images, they still improved the results. This is evident in the differences between S1 and S2, S3 and S4. The results of S2 are currently under embargo until they can be validated and explained with the help of visualisation tools.
- A more robust performance is achieved for a small number of images from the same domain in the source of the TL than using a large number of images from different domains, as shown in the differences between S2 and S3.
- S4 achieved the best results because ImageNet TL can speed up convergence, while the TL image in the domain can help alleviate the domain mismatch problem.
  The success of Scenario 4 can be attributed to its unique approach, which combines hybrid TL and relevance to a specific domain, which leads to a high level of generalisation with a better representation of the features. Moreover, its performance is further enhanced by the feature fusion technique employed. This combination enables the model to leverage pre-existing knowledge while adapting specifically to the target dataset. As a result, it performs better in detecting abnormalities in shoulder X-ray images.

**Table 5:** Results of DL models in the MURA dataset-shoulder task test set.

Evaluation Metric (%)	DenseNet201			
-	S1	S2	S3	S4
Accuracy	57.5	69.4	72.8	73.8
Specificity	97.5	66.8	83.8	85.6
Recall	16.5	78.6	61.5	61.8
Precision	86.7	73.2	78.8	80.7
F1 _*Score*_	27.7	72.2	69.0	70.0
Cohen’s kappa	14.2	38.7	45.4	47.6
-	ResNet101			
Accuracy	57.0	65.7	72.6	74.7
Specificity	46.6	78.6	80.7	81.0
Recall	67.6	52.5	64.3	68.3
Precision	55.2	70.5	76.5	77.8
F1 _*Score*_	60.8	60.2	69.9	72.8
Cohen’s kappa	14.2	31.2	45.1	49.4
-	NasNetLarge			
Accuracy	53.6	67.3	71.2	72.4
Specificity	70.1	68.4	76.1	80.0
Recall	36.6	66.1	66.1	64.7
Precision	54.5	67.1	73.0	75.9
F1 _*Score*_	43.8	66.6	69.4	69.9
Cohen’s kappa	6.89	34.6	42.3	44.8

### Part 2: Experimental assessment of deep-feature fusion

**Fig 14:**
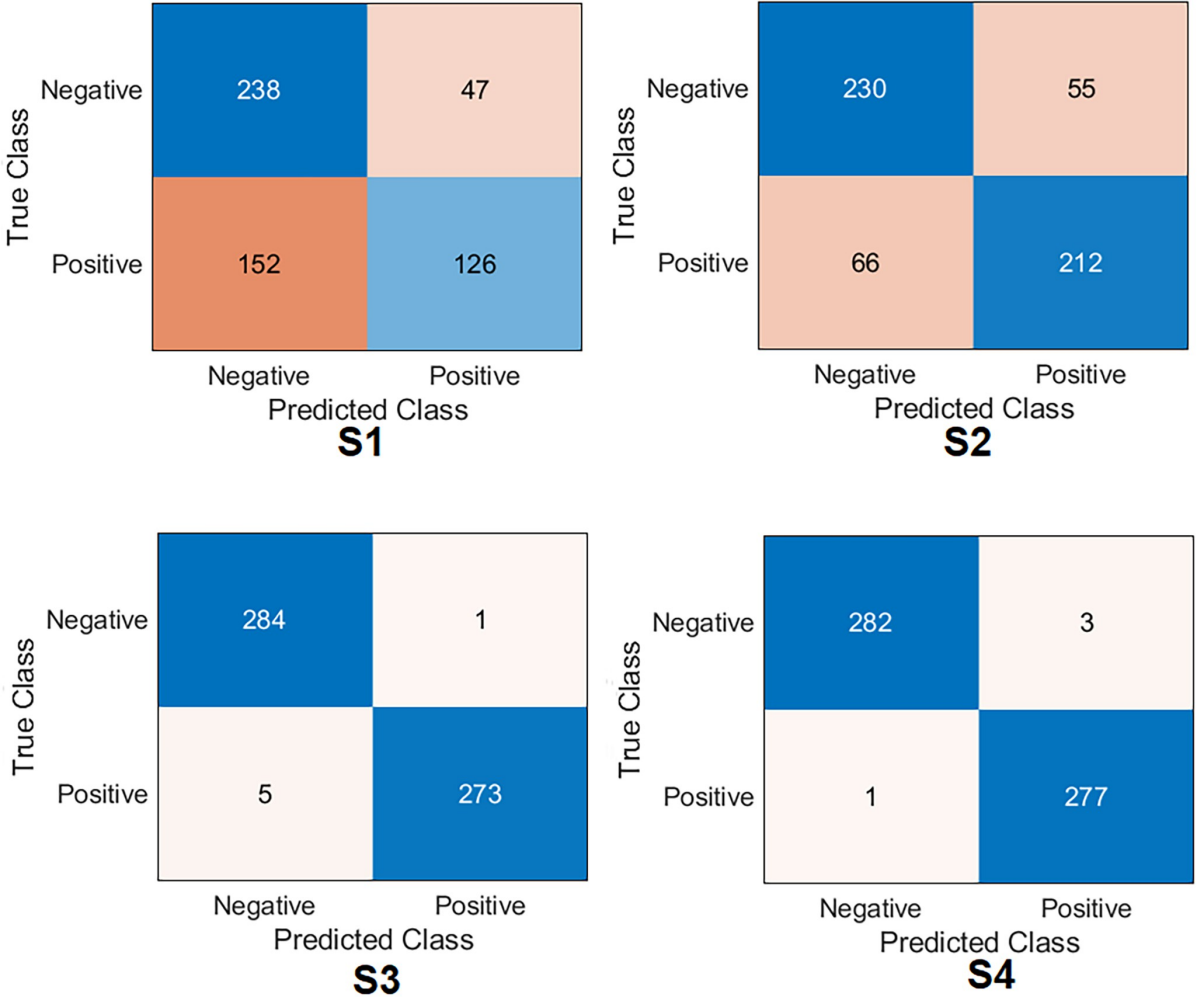
Confusion matrix of the feature fusion with logistic regression on the test set with four training scenarios.

**Fig 15:**
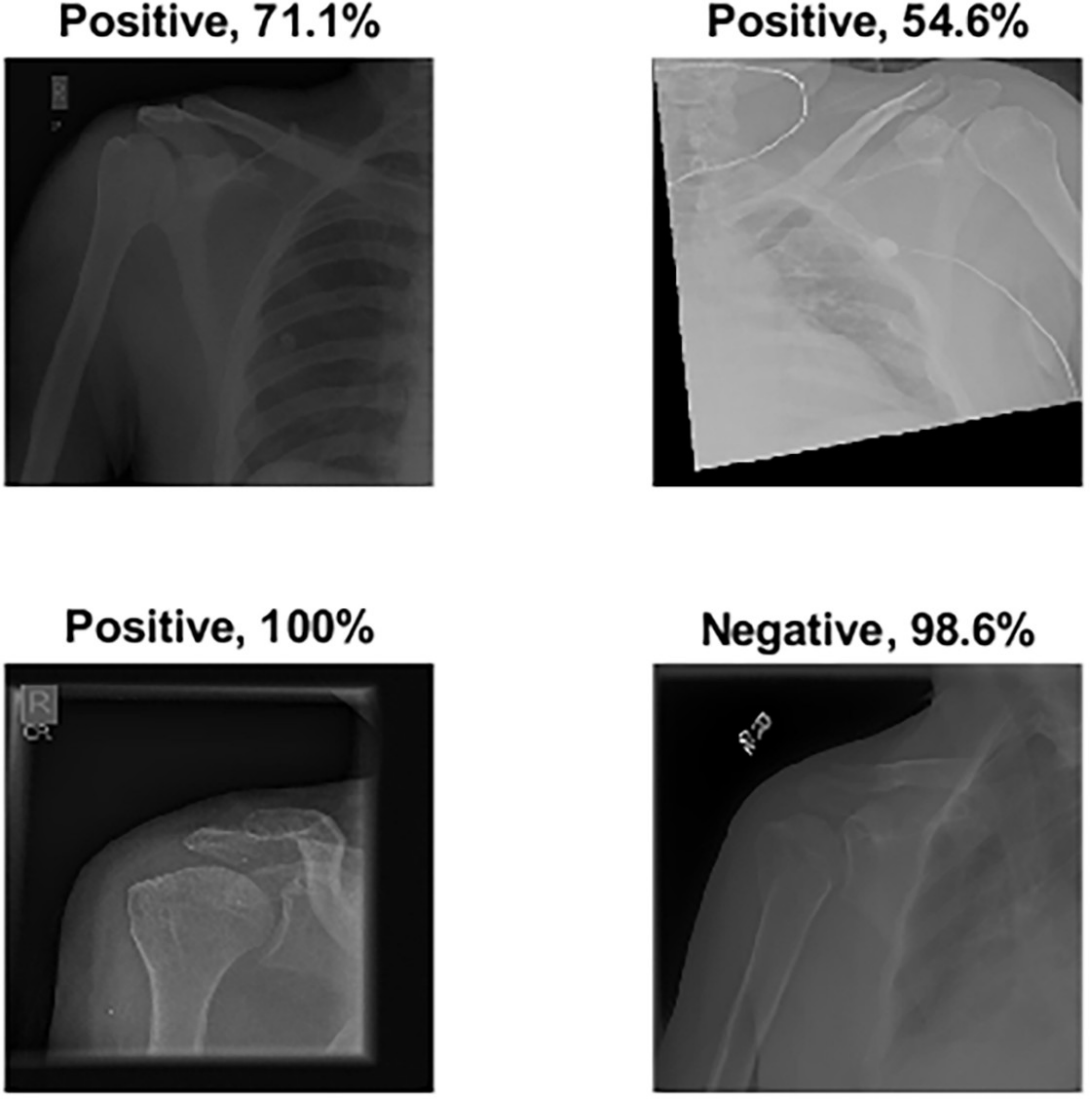
Misclassified samples from S4.

Several ML classifiers have been trained and tested with extracted features from seven models, including Decision Tree, Linear Discriminant, Naive Bayes, SVMs, K-Nearest Neighbour, Logistic Regression, and Neural Networks. The seven models have been used to extract the features of the four training scenarios. The results of the classifiers were very close. The logistic regression results are reported to show high performance in all scenarios. The confusion matrix was first calculated for all scenarios with Logistic Regression, as demonstrated in Fig 14. Surprisingly, the results listed in Table 6 show that S4 and S3 improved the results significantly compared to S1 and S2. S4 obtained an accuracy of 99.2%, a specificity of 98.9%, a recall of 99.6%, a precision of 98.9% and *F*1_*score*_ of 99.2%. At the same time, S3 obtained an accuracy of 98.9%, specificity of 99.6%, recall of 98.2%, precision of 99.6% and *F*1_*score*_ of 98.9%. The four samples that S4 misclassified are shown in Fig 15.

**Table 6:** Results of the fusion of features with the logistic regression classifier on a MURA dataset-shoulder task test set.

-	S1	S2	S3	S4
Accuracy	64.6	78.5	98.9	99.2
Specificity	83.5	80.7	99.6	98.9
Recall	45.3	76.2	98.2	99.6
Precision	72.8	79.4	99.6	98.9
F1 _*Score*_	55.8	77.8	98.9	99.2
Cohen’s kappa	28.9	56.9	97.8	98.5

On the other hand, S1 and S2 have achieved lower results than S3 and S4. S2 obtained an accuracy of 78.5%, a specificity of 80.7%, a recall of 76.2%, a precision of 79.4% and *F*1_*score*_ of 77.8%. However, S1 obtained an accuracy of 64.6%, a specificity of 83.5%, a recall of 45.3%, a precision of 72.8%, and *F*1_*score*_ of 55.8%.

Lastly, regarding Cohen’s kappa, S4 obtained the highest value by achieving 98.5%. With very little difference, S3 was placed in the second position, achieving 97.8%. Cohen’s kappa value was reduced with S2 and S1 by obtaining 56.9% and 28.9%, respectively.

Several conclusions can be highlighted from Table 6:

- The high results for S3 and S4 can be attributed to the excellent features that the models extracted. This proves that the proposed TL method effectively enabled the models to distinguish between different classes and extract fully descriptive features.
- It has been demonstrated through S1 and S2 that addressing the problems of data scarcity and domain mismatch is necessary for feature fusion to enhance performance.
- The same-domain TL with feature fusion helps to extract a wide range of features. It also allows to avoid overfitting with high generalisation. It is clear from the results of other classifiers with S4 that the SVMs achieved an accuracy of 99.1%, 99.0% with Decision Tree, 98.3% with Linear Discriminant, 98.6% with Naive Bayes, 99.0% with K-Nearest Neighbour and 97.2% with Neural Networks.

### Visualisation techniques

**Fig 16:**
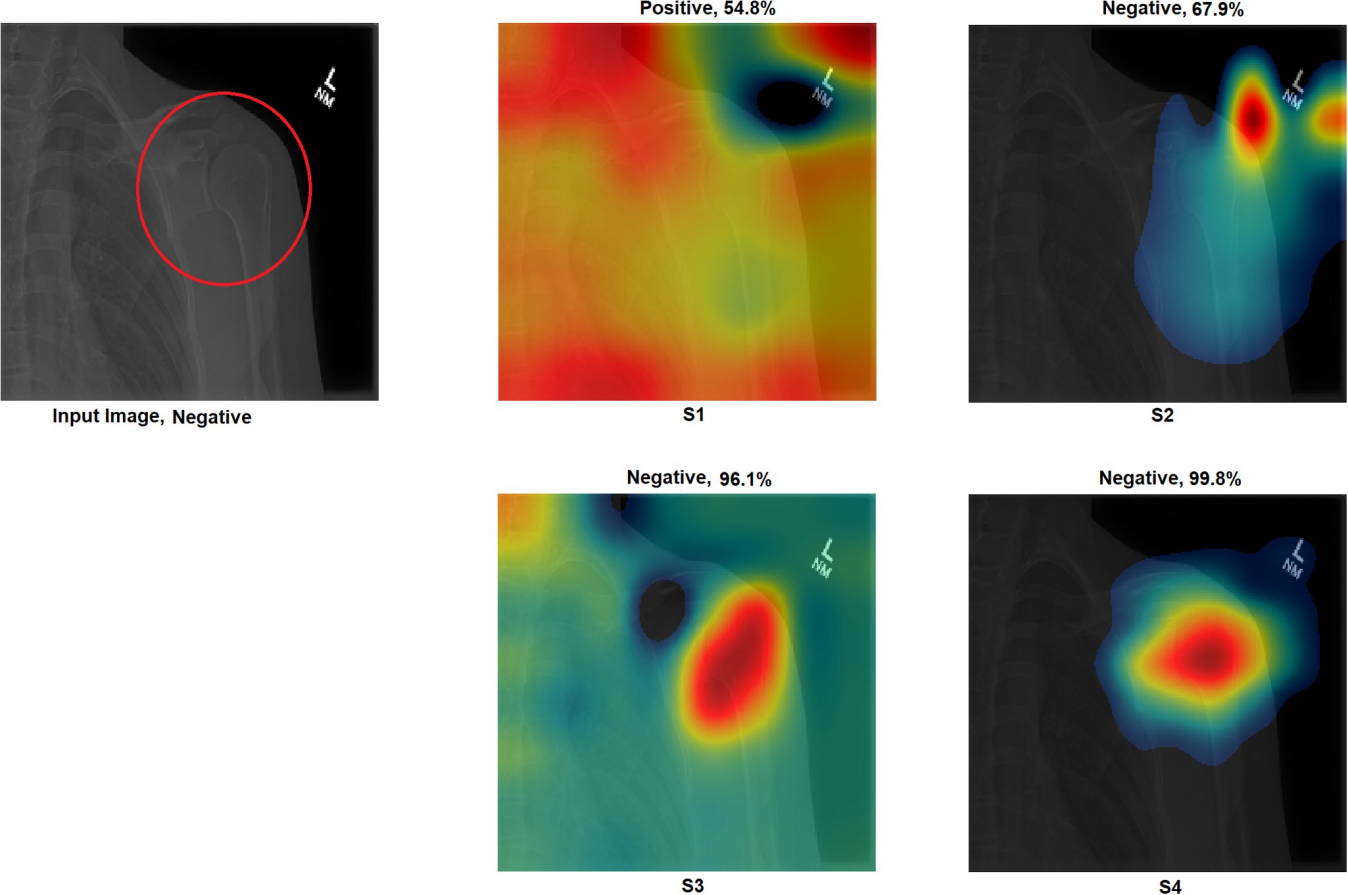
Grad-CAM and Score- Grad-CAM for shoulder X-ray image. The correct classification is Negative. The ROI is the red circle that a domain expert has marked.

**Fig 17:**
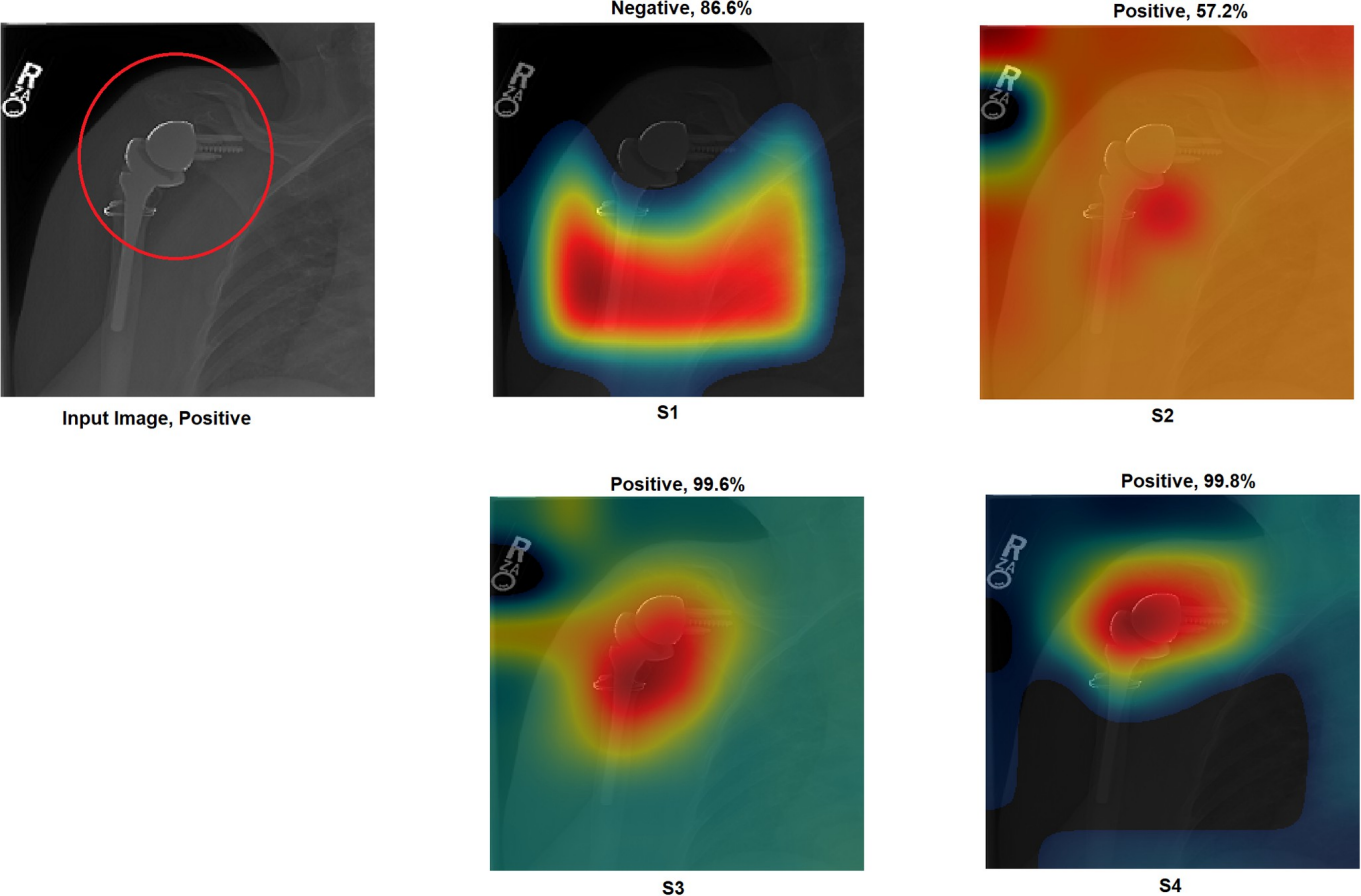
Grad-CAM and Score- Grad-CAM for shoulder X-ray image. The correct classification is Positive. The ROI is the red circle that a domain expert has marked.

**Fig 18:**
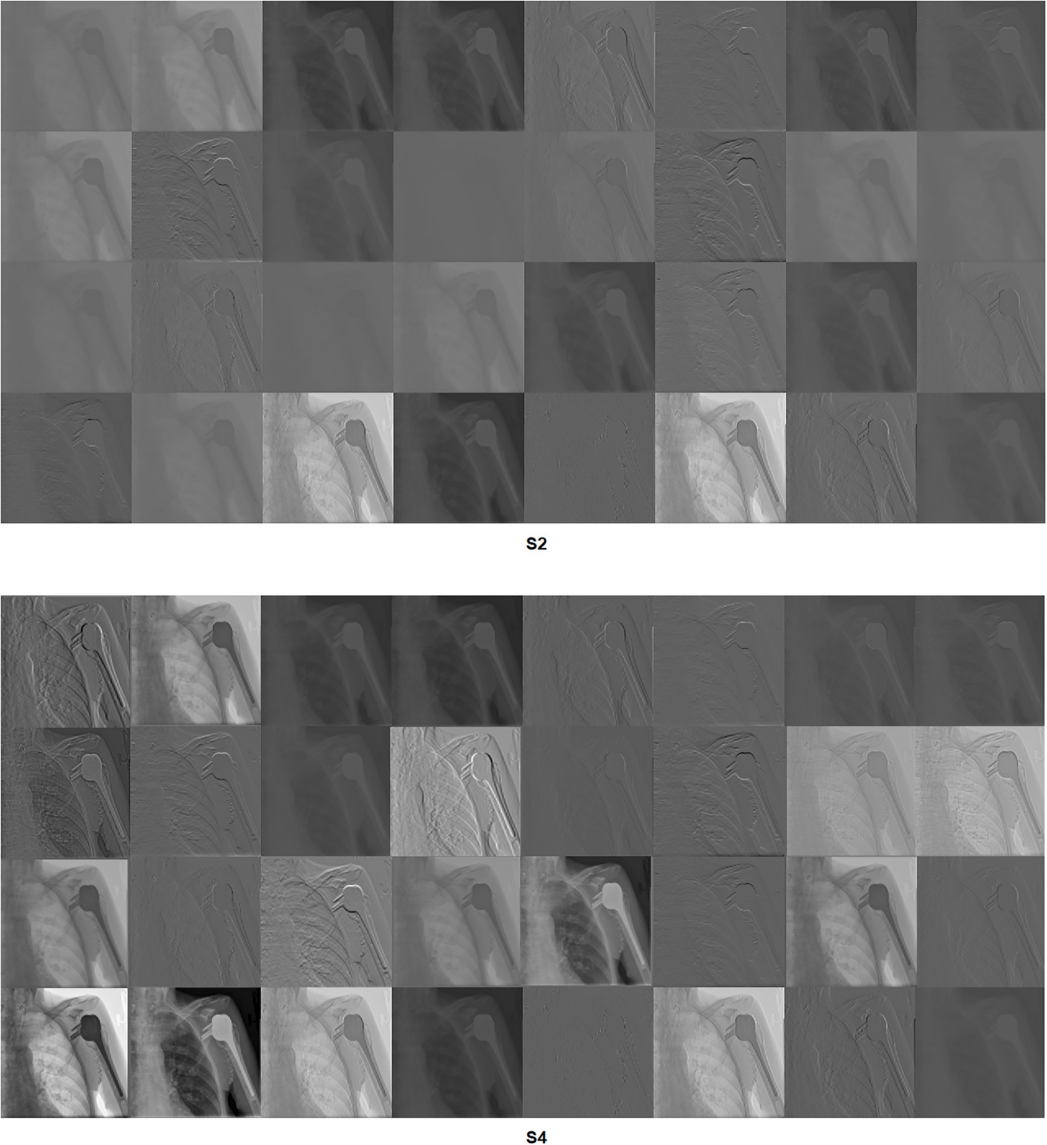
Learned filters of the first convolutional layer.

**Fig 19:**
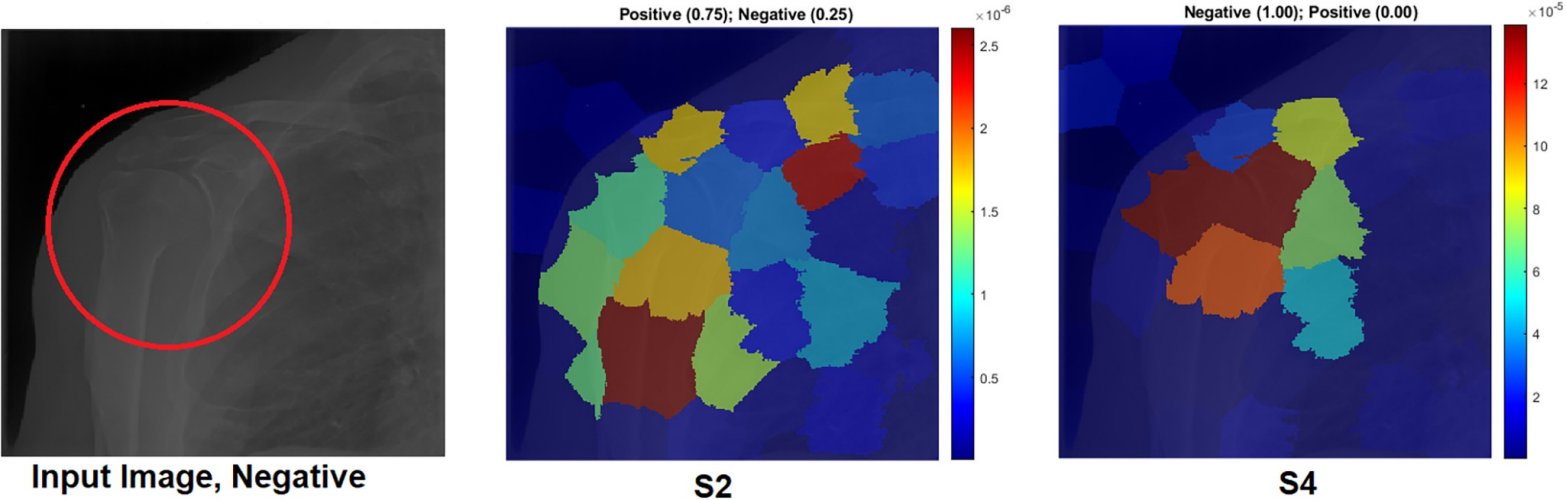
LIME and Score-LIME for shoulder X-ray image. The correct classification is Negative. The ROI is the red circle that a domain expert has marked.

To explain the “black box” of DL models with the four training scenarios, the following three visualisation techniques have been adopted:

1. Grad-CAM: In this section, two examples have been presented. The first one (Fig 16) presents a negative sample with all four scenarios. It shows that the model with S1 misclassified the test sample, and the heat map focuses on areas outside the region of interest (ROI). However, the model with S2 classified the sample correctly but with a low confidence value, while the related heat map indicates that a great deal of attention was paid to areas outside of the ROI. With S3 and S4, the model correctly classified the sample with a high confidence value, while the related heat map indicates a focus on ROI.
  The second (Fig 17) presents a positive sample where it shows the same scenario as Example 1 (Fig 16). Fig 17 shows that with S3 and S4, the model correctly classified the sample with a high confidence value and with the heat map aiming at ROI. The model with S2 correctly classified the sample but with a low confidence value, and the heat map shows that the model is looking over the image. Lastly, the model with S1 misclassified the test sample, and the heat map focuses on areas outside the ROI.
  These two examples show that the proposed approach significantly helped improve the results. On the other hand, S2 showed a correct prediction, but it cannot be trusted due to low confidence and focus outside of ROI. Lastly, the two samples have been misclassified with an out-of-the-ROI focus when considering S1.
  For the remainder of this study, we focus on the comparison between S2 and S4 due to the following reasons: i) regarding S1, the results are low and inaccurate, so it is dismissed; ii) S3 and S4 have almost the same outcome, and both follow almost the same procedure; iii) most of the previous scenarios used the same technique of S2 which maintains fairness; iv) S2 and S4 are the same concepts except that our proposal of TL is added to S4; v) regarding S4, the highest results are achieved.
2. Activation Visualisation:
Fig 18 compares S2 and S4 in terms of features learnt by the model from the first convolutional layer. Due to the proposed TL, the model captured good features with S4.
3. LIME:
Fig 19 compares S2 and S4 in terms of LIME and Score-LIME. According to S2, the model has wrongly predicted the test sample where the high-intensity area is out of the ROI. On the other hand, the model with S4 correctly predicted the input sample with a confidence value of 100%. The LIME shows that the model identified the ROI as the highest intensity value. This example proves the effectiveness of the proposed approach by changing the wrong prediction to the correct one.

### Comparison against the state-of-the-art

This section compares our proposal against the recent methods in the field working on the same dataset.

The results listed in Table 7 are the highest in the shoulder task considering the MURA dataset. It is remarkable how the proposed approach outperformed all the methods for several reasons. First, in the case of Uysal’s method [30], there were 26 DL-based pre-trained models trained and tested. Furthermore, two ensemble learning models (EL1 and EL2) were proposed, and these achieved the highest results compared to the 26 individual models and most of the methods tested. Although Uysal’s method [30] used ensemble learning models, its main drawback was due to the extracted features, which are not good enough to distinguish between classes. This proves that the TL proposal is more suitable for the learnt features than the TL from the ImageNet dataset.

**Table 7:** Comparison against the state-of-the-art considering the MURA dataset test set for the shoulder detection task.

Ref.	Evaluation Metric (%)				
-	Accuracy	Recall	Precision	F1 _*Score*_	Cohen’s kappa
Uysal, F., et al. [30] (EL1), 2021	84.5	81.6	86.3	84.5	69.0
Uysal, F., et al. [30] (EL2), 2021	84.7	84.5	85.0	84.5	69.4
He, Minliang, et al. [43], 2021	85.0	83.0	86.0	-	70.0
**Ours (S3)**	98.9	98.2	99.6	98.9	97.8
**Ours (S4)**	99.2	99.6	98.9	99.2	98.5

The main criticism against the state-of-the-art methods is that they utilised a small dataset for training deep models and used mismatched features from the ImageNet dataset to overcome data scarcity. The size of the adapted dataset is notably tiny, which could result in the risk of overfitting with less generalisation. However, our proposal has demonstrated improved performance in dealing with these issues due to the models learning a wide range of relevant features, which can be shown from the results considering S3 and S4.

### Comparison against the orthopaedic surgeons

**Fig 20:**
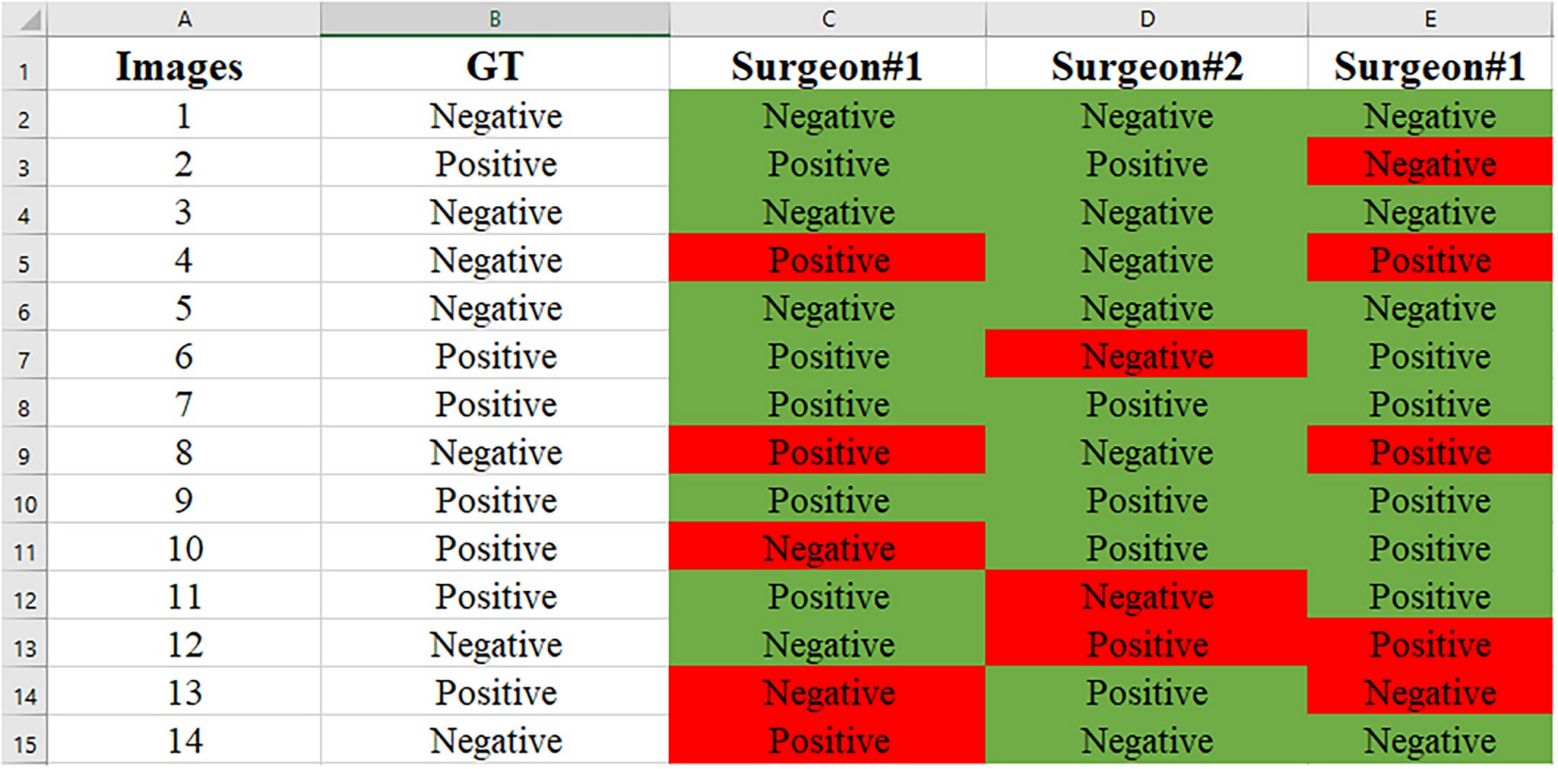
Sample recording of the results obtained by surgeons.

Three orthopaedic surgeons from Greenslopes Private Hospital-Brisbine Australia were invited to classify the test set for the MURA dataset-shoulder task. The experimental setup for the surgeon’s diagnosis on the test set (285+ 278) was as follows:

- Each image within the test set was uniquely identified by assigning them distinctive numerical labels.
- The images representing both classes were subsequently subjected to randomisation to ensure an unbiased distribution.
- A comprehensive record was maintained in an Excel spreadsheet, documenting the numerical label of each image along with its corresponding ground-truth classification.
- Each surgeon was individually arranged in a dedicated private space where they evaluated every image displayed on a large screen, providing their diagnostic evaluation. It should be noted that each surgeon performed his evaluations on separate days to avoid possible bias or influence from other participants.
- To ensure accuracy in data collection, an additional individual equipped with an Excel spreadsheet was responsible for meticulously recording the diagnostic responses provided by the surgeons.
- After collecting diagnostic results, they were cross-referenced with ground truth information to calculate correct and misclassified samples (see Fig 20).
- It took each surgeon almost 2 hours and 30 minutes to predict the outcomes.

**Fig 21:**
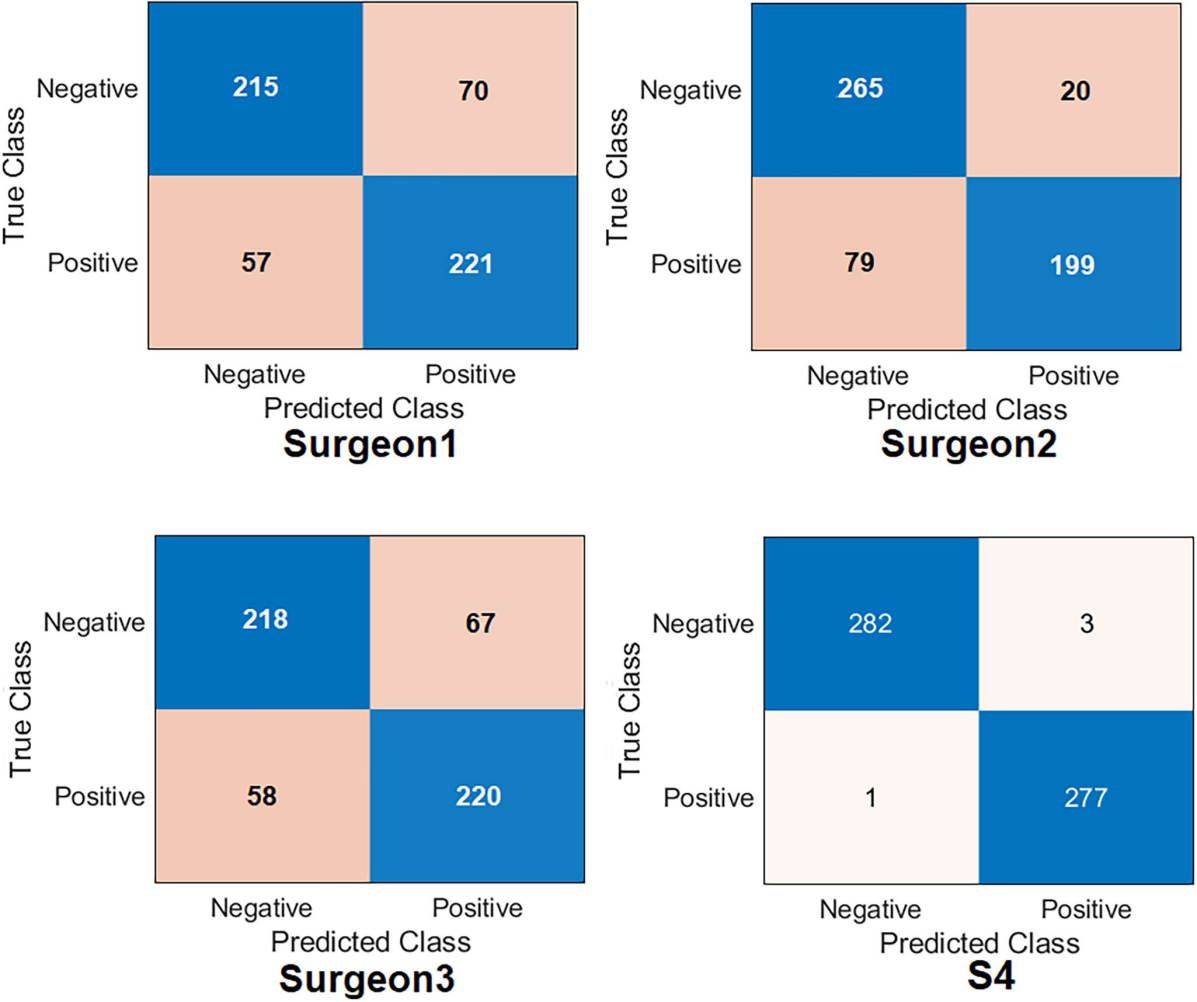
Confusion matrix of the orthopaedic surgeons compared to S4-Fusion.

Fig 21 and Table 8 report the results of orthopaedic shoulder specialists. Surgeon#2 has shown the highest score compared to the other two clinicians by obtaining an accuracy of 82.4%, specificity of 90.87%, recall of 77.03%, the precision of 92.9%, and *F*1_*score*_ of 84.2%. The other two surgeons displayed a very similar performance: Surgeon#1 obtained an accuracy of 77.4%, specificity of 75.4%, recall of 75.9%, the precision of 79.0%, and *F*1_*score*_ of 77.2%; Surgeon#3 obtained an accuracy of 77.8%, specificity of 76.6%, recall of 78.9%, precision of 76.4%, and *F*1_*score*_ of 77.7%. Regarding Cohen’s kappa, surgeon#2 obtained the highest value compared to other surgeons by reaching 64.7%, surgeon# 3 reached 55.6%, and surgeon#1 reached 54.9%. It is impressive that the proposed DL model outperformed the three experts. This test aimed to highlight that the predictions of DL technologies inspire surgeons with more confidence to use them in their daily practice.

**Table 8:** Results of the comparison with orthopaedic surgeons against DL.

Evaluation Metric (%)	Surgeon#1	Surgeon#2	Surgeon#3	Ours (S4)
Accuracy	77.4	82.4	77.8	**99.2**
Specificity	75.4	90.87	76.6	**98.9**
Recall	75.9	77.03	78.9	**99.6**
Precision	79.0	92.9	76.4	**98.9**
F1 _*Score*_	77.2	84.2	77.7	**99.2**
Cohen’s kappa	54.9	64.7	55.6	**98.5**

The MURA dataset was intentionally collected with the primary focus on not defining abnormalities, and these anomalies were deliberately ignored during the image evaluation process. The rationale behind this approach is to develop a tool that can assist specialists when they encounter similar scenarios in emergencies, for instance, or when the images provided are not ideal in terms of resolution and availability of different views, among others. Throughout the experiment, orthopaedic specialists shared some of the abnormalities they encountered when diagnosing the images. These abnormalities encompass:

- presence of fracture
- present of deformity in the bones
- implants not positioned properly
- signs of arthritis
  - narrowed joint
  - osteophytes (bony spurs)
  - sclerosis (increase in density on the edge of the bones that form the joint)
  - subchondral cysts (small fluid-filled spaces near the edge of the bones that form the joint)
- presence of lucency (less dense or dark areas) around the implants
- presence of abnormal lesions in the bone

This approach of intentionally omitting predefined abnormalities creates a more realistic and challenging dataset, mirroring the complexities that medical professionals face in real-world scenarios. Training models on such data aims to enhance their ability to help specialists make accurate diagnoses, even in cases where an evident abnormality is not immediately apparent. This approach accounts for the multifaceted nature of clinical decision-making and equips models to handle diverse and complex situations effectively. The comparison with specialists highlights the urgent need for supportive tools to decide in emergency situations.

**Fig 22:**
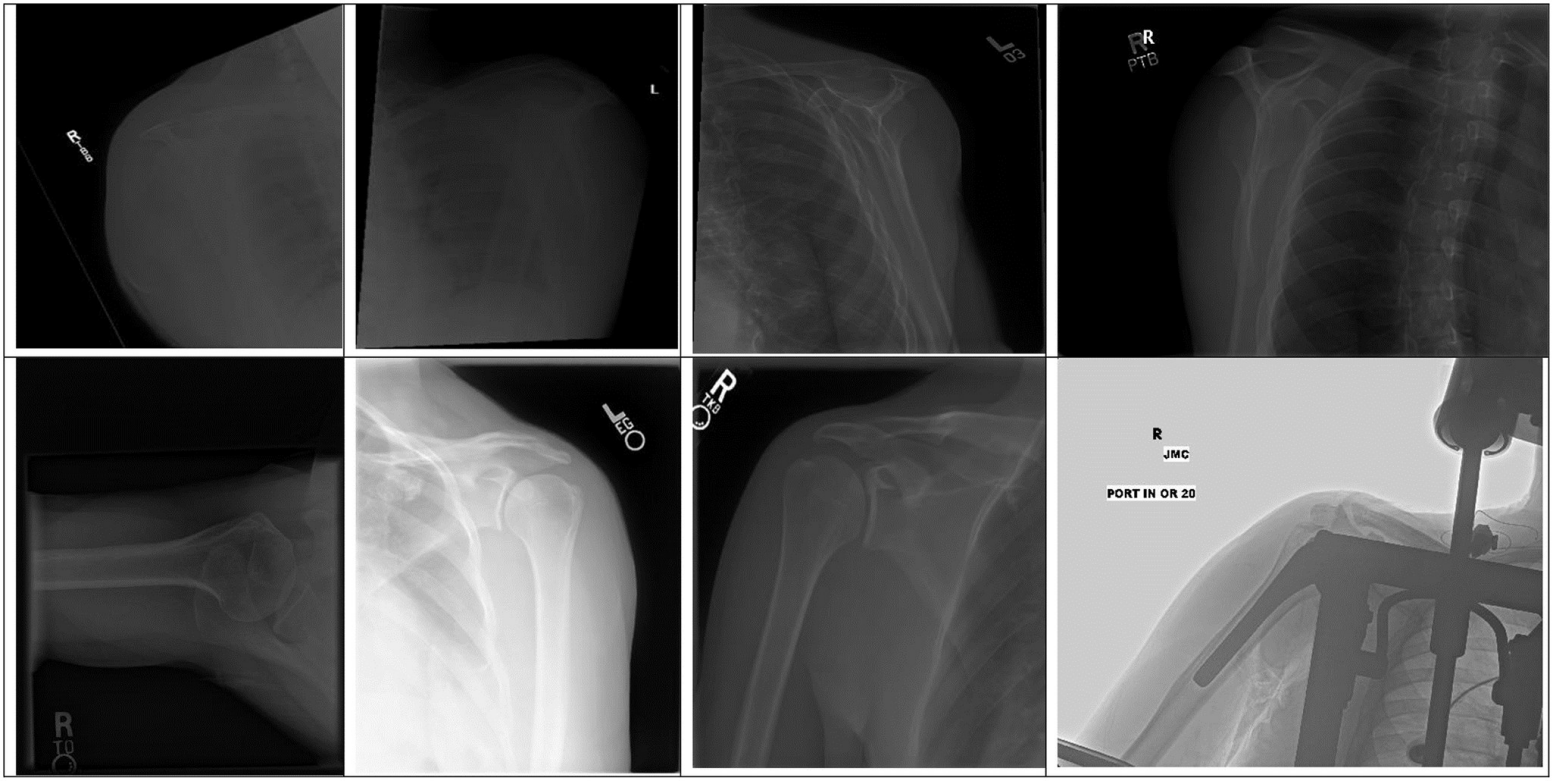
Misclassified samples by the surgeons were divided into two rows, with the first row indicating negative class and the second-row indicating positive class.

Surgeons may misclassify specific images due to various factors. Among the main reasons is the many images’ poor quality, making it difficult for surgeons to evaluate them accurately. Inadequate lighting or low contrast can obscure bony structures, making diagnosis difficult. For instance, in Fig 22, the images in the first row were labelled positive, but the ground truth indicates that they are negative. This is likely due to the poor quality of the images, which made it difficult for surgeons to assess the bony structures accurately.

The second reason is that the specialists who evaluated the X-ray images were orthopaedic surgeons. Typically, they would need to evaluate the images in more than one view to make an accurate diagnosis. Furthermore, the evaluation of the X-ray images would often require clinical correlation to confirm whether the image is abnormal. This means that specialists would compare what they see in the pictures with patient complaints, age, symptoms, and physical examination. In contrast, the specialists who evaluated the images in the original dataset were radiologists. They are more accustomed to assessing images in isolation and may not always require clinical correlation to make a diagnosis.

Lastly, since the specialists who labelled the images were orthopaedic surgeons who are shoulder subspecialists, they are very critical of the shoulder joint, so even minor changes can be labelled abnormal. For example, some images were labelled positive because the specialists saw signs of mild arthritis, which were sclerosis on the glenoid rim and a slight narrowing of the glenohumeral joint, as shown in Fig 22, second row. However, to confirm whether these were positive, specialists usually need to verify with another X-ray view and correlate with clinical symptoms.

In [4], three radiologists were invited to evaluate the shoulder task of the MURA dataset, and their evaluation was compared to our proposed DL model as reported in Table 9. Again, our proposal significantly outperformed the experts’ results.

**Table 9:** Kappa scores of three radiologists reported in reference [4] compared to our results for the shoulder task.

	Rad#1	Rad#2	Rad#3	Ours (S4)
Cohen’s kappa	86.4	79.1	86.4	**98.5**

The DL model overcame the issues faced by orthopaedic surgeons in the shoulder task of the MURA dataset by excelling in several key areas. DL models are adept at automatically extracting relevant features from images, even when they are low quality, and can simultaneously process multiple views of an image. They offer consistent and objective evaluations, have been trained on diverse datasets, can generalise to new data, and provide rapid predictions. This versatility, speed, and adaptability allowed the DL model to outperform human experts, demonstrating its potential to enhance the accuracy and efficiency of medical image analysis and diagnostics, especially in challenging and less-than-ideal clinical scenarios.

In the S1 and S2 Appendices, we have presented some test samples that were analysed using Grad-CAM. We conducted a small experiment to validate the results and consulted with domain experts. During the consultation, we asked them specific questions about the highlighted regions, such as “Do these regions correspond to what you would expect in this context?” and they answered positively. We also asked if there were any anomalies or unexpected findings, and they replied yes to some of them. Furthermore, we enquired whether these regions aligned with known patterns or features in the data, and they answered yes.

### Robustness of our proposal

**Fig 23:**
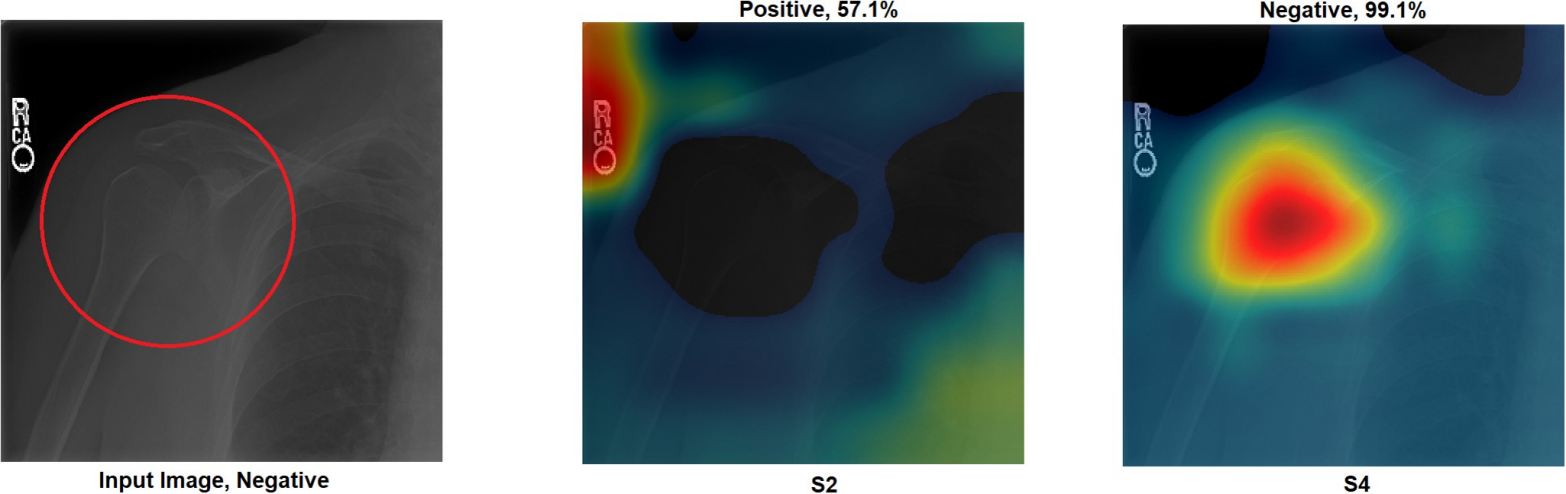
Grad-CAM and Score- Grad-CAM for shoulder X-ray image. The correct classification is Negative. The ROI is the red circle that a domain expert has marked.

**Fig 24:**
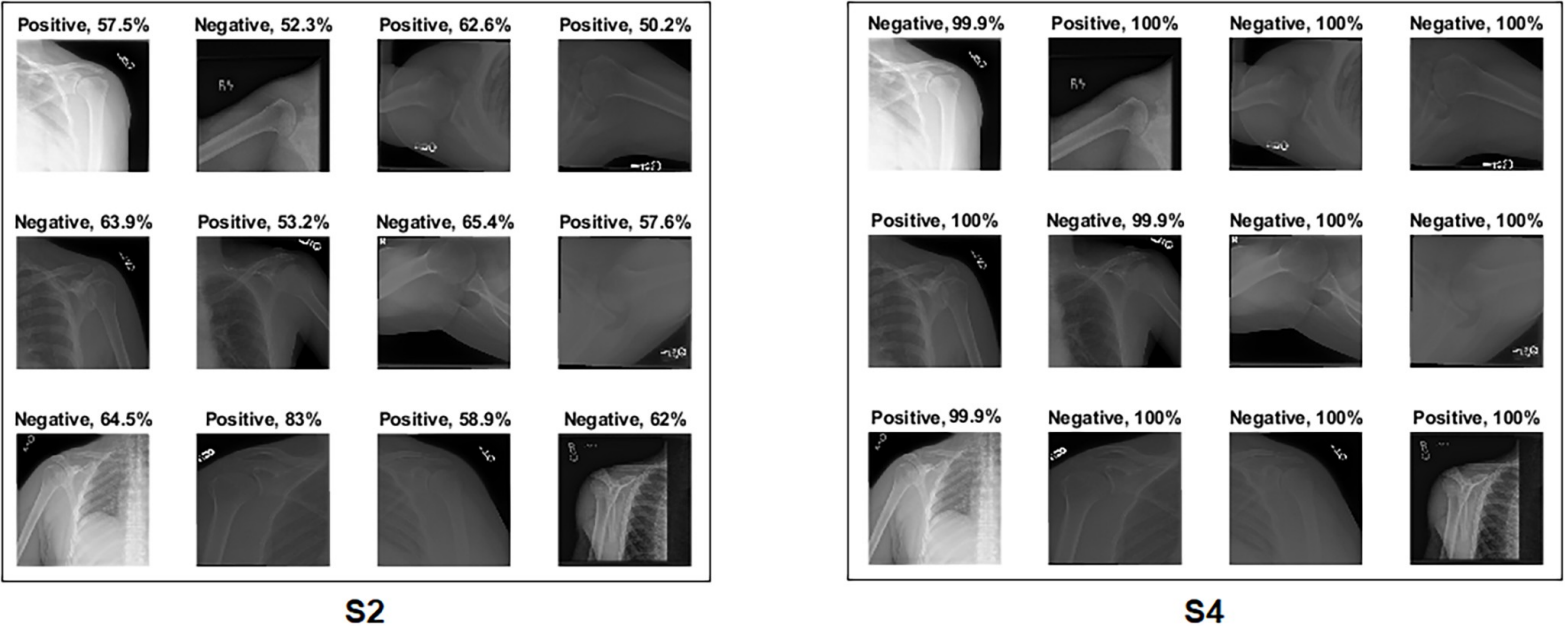
Comparison between S2 and S4, where S2 misclassified the samples and S4 correctly classified them.

**Fig 25:**
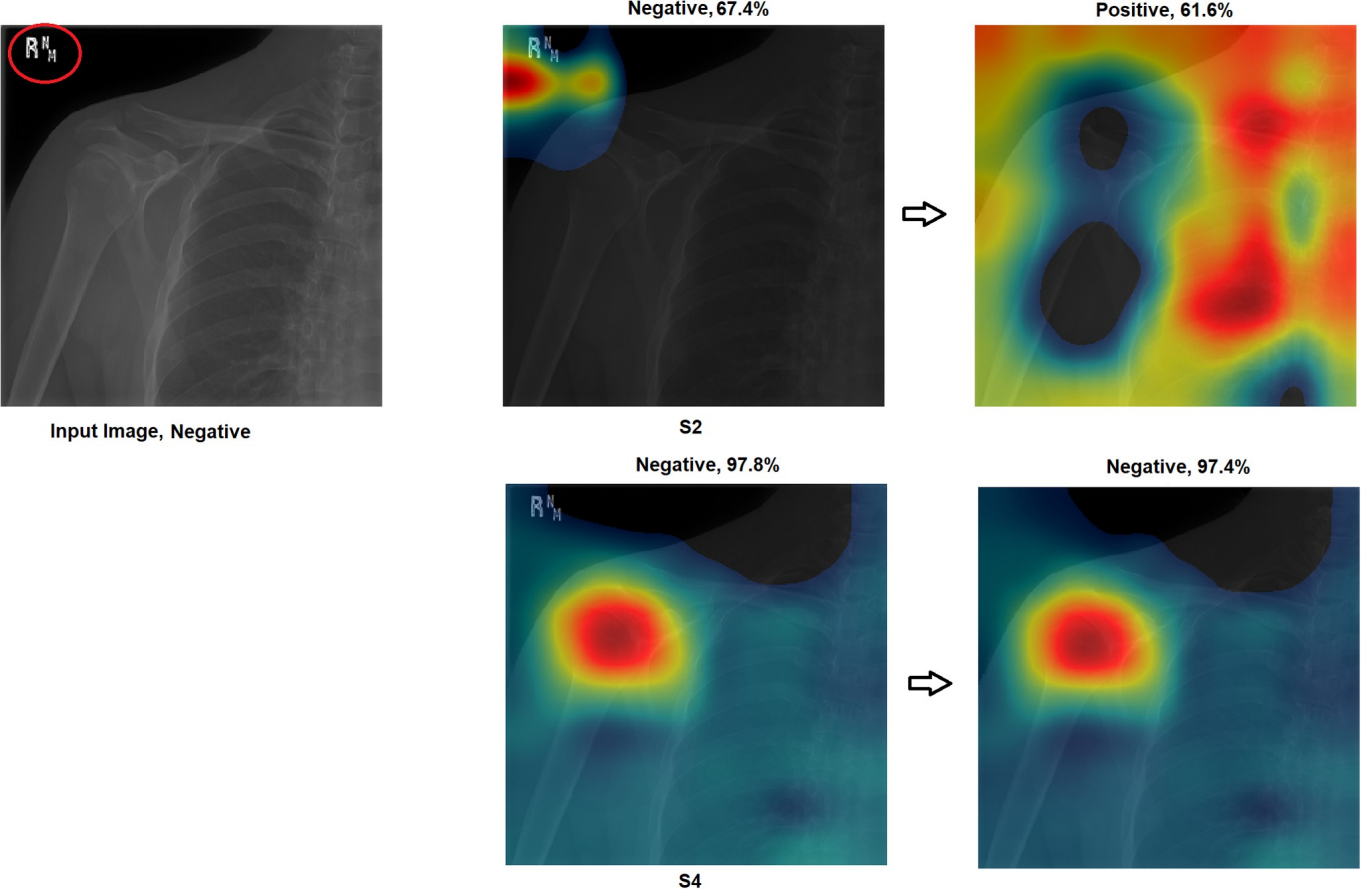
Effect of any change by removing the written letters in the red circle. The correct classification is Negative. The ROI is the red circle that a domain expert has marked.

**Fig 26:**
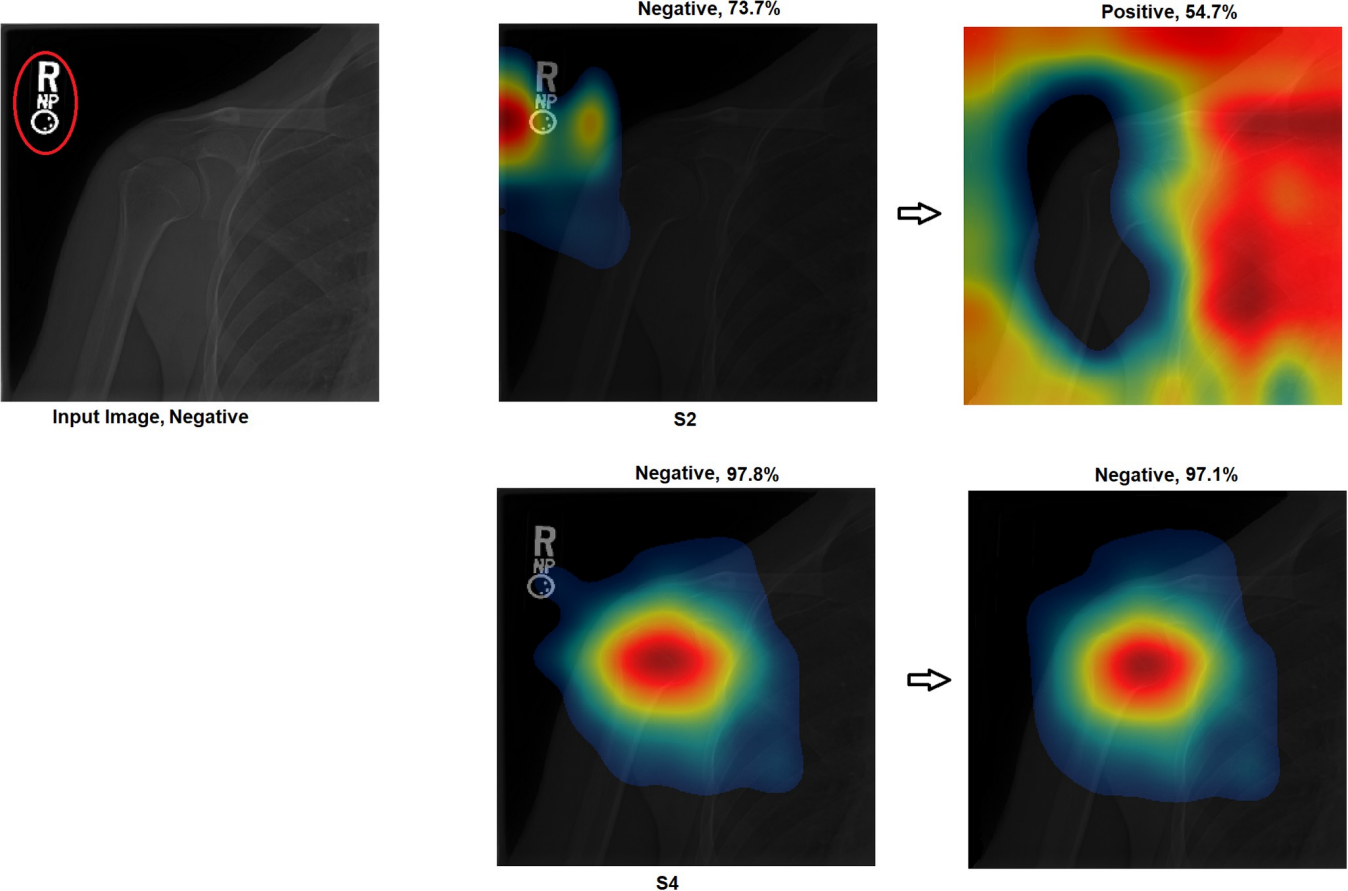
Effect of any change by removing the written letters in the red circle. The correct classification is Negative.

**Fig 27:**
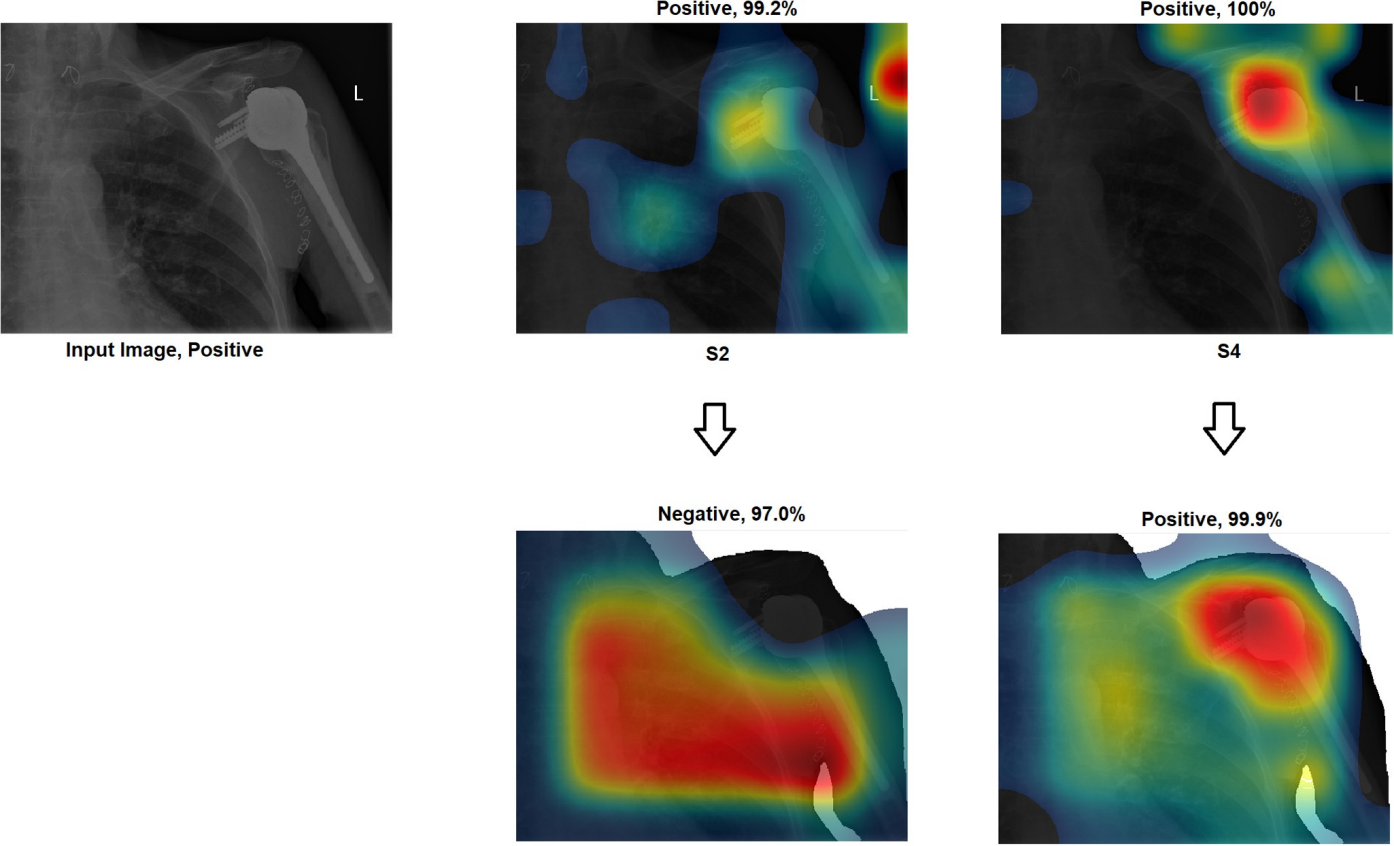
Comparison between S2 and S4, where the correct classification is positive.

**Fig 28:**
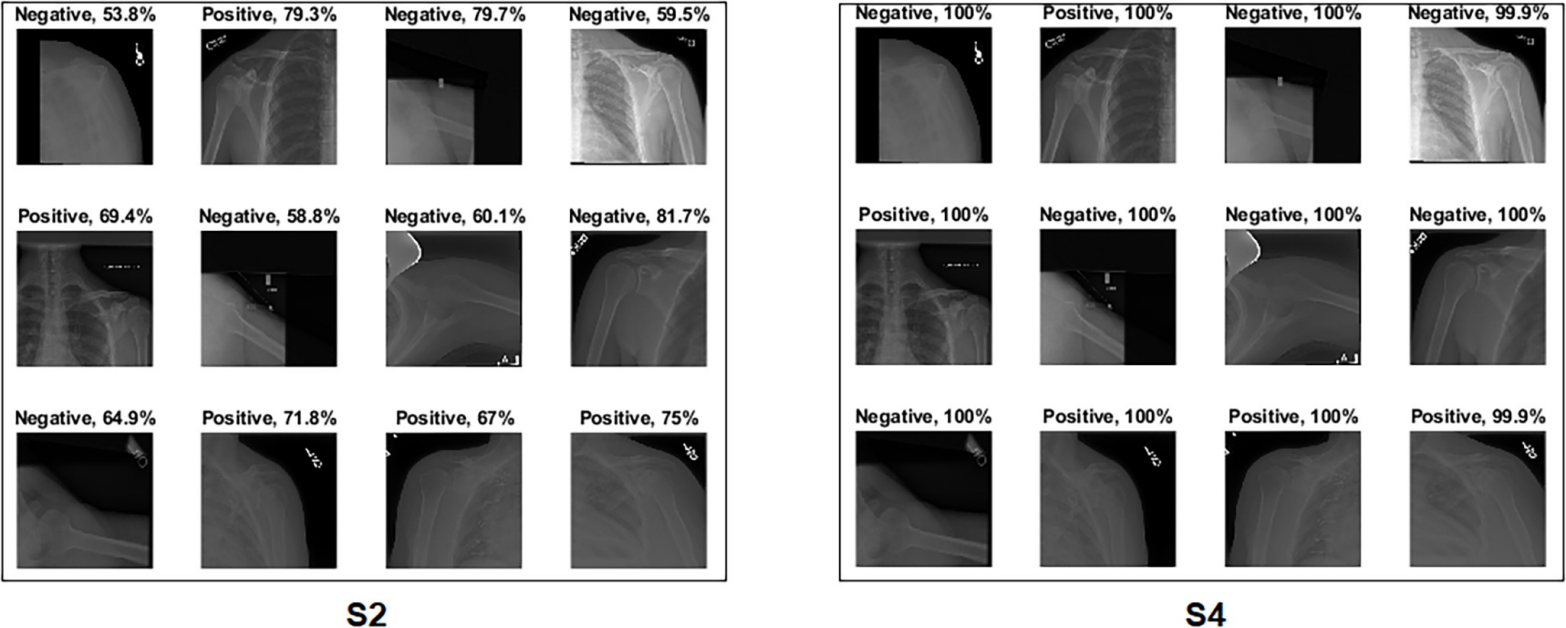
Comparison between S2 and S4, where S2 and S4 correctly classified the samples but with different confidence score values.

This section aims to validate the robustness of our approach as follows:

- Outcome improvement:
  Figs 23 and 24 show the comparison between S2 and S4, where S4 has improved the prediction from wrong to correct with a high confidence value. Both figures showed that the proposed approach (S4) has significantly improved performance with an accurate identification of ROI. From Fig 23, S4 identified the right ROI to decide, while S2 only pointed out the ROI.
- Test against change:
  Our approach (S4) was tested against different changes to prove its robustness. Figs 25 and 26 show that the performance of S2 changes with minor changes, such as removing the written letters in the red circle. The prediction changed from correct to incorrect before and after the change and was aimed outside the ROI. On the other hand, S4 showed performance stability by correctly predicting samples with a high confidence value and accurately identifying ROI.
- Confidence assessment:
  - High score
    Fig 27 shows an interesting case where S2 correctly classified the sample with a high confidence value, but aimed outside the ROI. Although the confidence value is high, it cannot be trusted, as the Grad-CAM visualisation points to the opposite. When the background was removed, the sample was wrongly classified with a high confidence value.
  - Low score
    Fig 28 depicts several test samples where S2 and S4 correctly classified them. S2 predicted samples with low confidence values, which cannot be trusted, as the model is uncertain about the sample, especially those with confidence values in the 50s. However, S4 showed a high confidence score value that can be trusted in the prediction.

## Conclusions

This paper presents a trustworthy DL framework for identifying abnormalities in shoulder radiography. Seven pre-trained ImageNet models were initially trained on in-domain X-ray images (such as elbow, finger, forearm, hand, humerus, and wrist) to overcome the domain mismatch between coloured nature images and greyscale X-ray images. The models were then trained on the target dataset for the shoulder task. This approach was compared with three different training scenarios, including training from scratch on the target dataset (S1), with ImageNet on the target dataset (S2), and with a new TL source compared to the target (S3). The results showed that when ImageNet was used as the base, training on the source of the TL and then on the target dataset (S4) was the best for the seven models when individually evaluated. The seven models trained with each scenario were used to extract features, combined to train six machine-learning classifiers. The proposed TL approach reduced the mismatch between the two domains, with S4 achieving an accuracy of 99.2%, outperforming other state-of-the-art methods. Furthermore, three visualisation tools were used to enhance the assessment of the reported results. Visual inspection of the results showed how the models using the proposed TL approach accurately identified the right area in the image to make the decision. The proposed DL significantly outperformed the results of three orthopaedic surgeons and radiologists. Our next step involves focussing on the classifier’s generalisation across various datasets.

## Supporting information

S1 Appendix Grad-CAM and Score- Grad-CAM analyses of shoulder X-ray image; negative test samples. 1.1 -Grad-CAM and Score- Grad-CAM analyses of Negative shoulder X-ray image. 1.2 -Grad-CAM and Score- Grad-CAM analyses of Negative shoulder X-ray image. 1.3 -Grad-CAM and Score- Grad-CAM analyses of Negative shoulder X-ray image. 1.4 -Grad-CAM and Score- Grad-CAM analyses of Negative shoulder X-ray image. 1.5 -Grad-CAM and Score- Grad-CAM analyses of Negative shoulder X-ray image. 1.6 -Grad-CAM and Score- Grad-CAM analyses of Negative shoulder X-ray image.1.7 -Grad-CAM and Score- Grad-CAM analyses of Negative shoulder X-ray image.
(ZIP)

S2 Appendix Grad-CAM and Score- Grad-CAM analyses of shoulder X-ray image; positive test samples. 2.1 -Grad-CAM and Score- Grad-CAM analyses of Positive shoulder X-ray image. 2.2 -Grad-CAM and Score- Grad-CAM analyses of Positive shoulder X-ray image. 2.3 -Grad-CAM and Score- Grad-CAM analyses of Positive shoulder X-ray image. 2.4 -Grad-CAM and Score- Grad-CAM analyses of Positive shoulder X-ray image. 2.5 -Grad-CAM and Score- Grad-CAM analyses of Positive shoulder X-ray image. 2.6 -Grad-CAM and Score- Grad-CAM analyses of Positive shoulder X-ray image.
(ZIP)

## Abbreviations

CNN: Convolutional Neural Network
DL: Deep learning
FN: False Negative
FP: False Positive
Grad CAM: Gradient-based Class Activation Heat Map
LIME: Locally Interpretable Model Independent Explanations
ML: Machine Learning
MURA: Musculoskeletal Radiographs
ROI: Region of Interest
TL: Transfer Learning
TN: True Negative
TP: True Positive
