# Supplementary figures and images for: Trustworthy deep learning framework for the detection of abnormalities in X-ray shoulder images
Source: PLoS One. 2024 Mar 11;19(3):e0299545. doi: 10.1371/journal.pone.0299545 (PMC10927121; doi:10.1371/journal.pone.0299545)

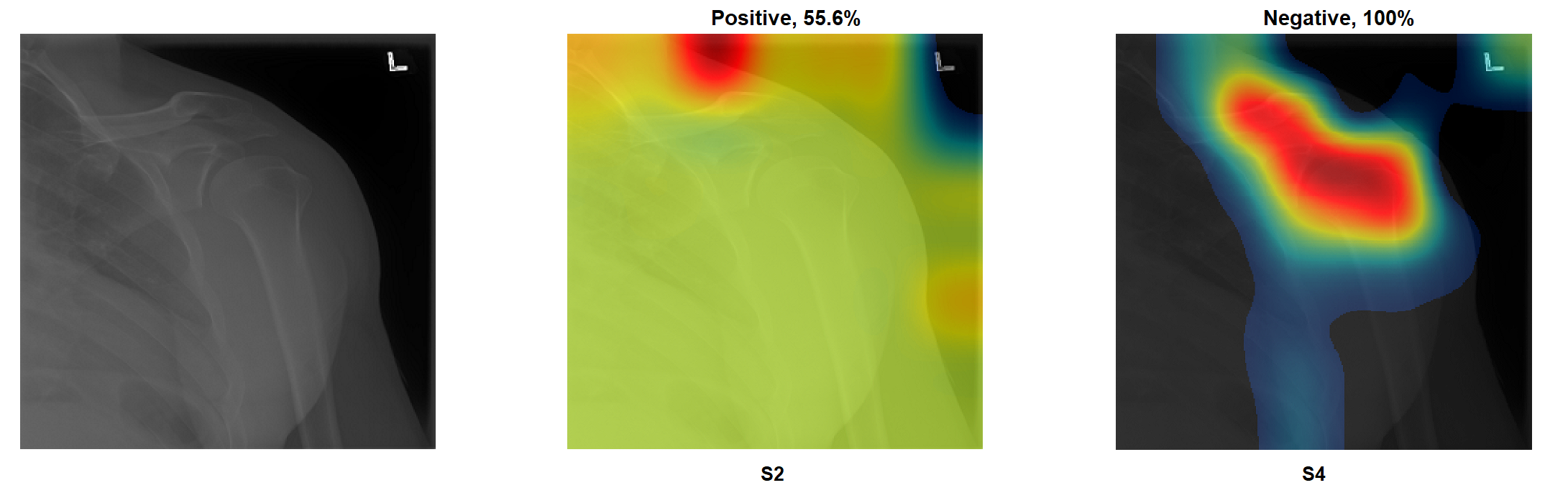

Supplement: S1 Appendix — 1.1 -Grad-CAM and Score- Grad-CAM analyses of Negative shoulder X-ray image. 1.2 -Grad-CAM and Score- Grad-CAM analyses of Negative shoulder X-ray image. 1.3 -Grad-CAM and Score- Grad-CAM analyses of Negative shoulder X-ray image. 1.4 -Grad-CAM and Score- Grad-CAM analyses of Negative shoulder X-ray image. 1.5 -Grad-CAM and Score- Grad-CAM analyses of Negative shoulder X-ray image. 1.6 -Grad-CAM and Score- Grad-CAM analyses of Negative shoulder X-ray image.1.7 -Grad-CAM and Score- Grad-CAM analyses of Negative shoulder X-ray image. (ZIP) [file pone.0299545.s001.zip › App1.7.png]

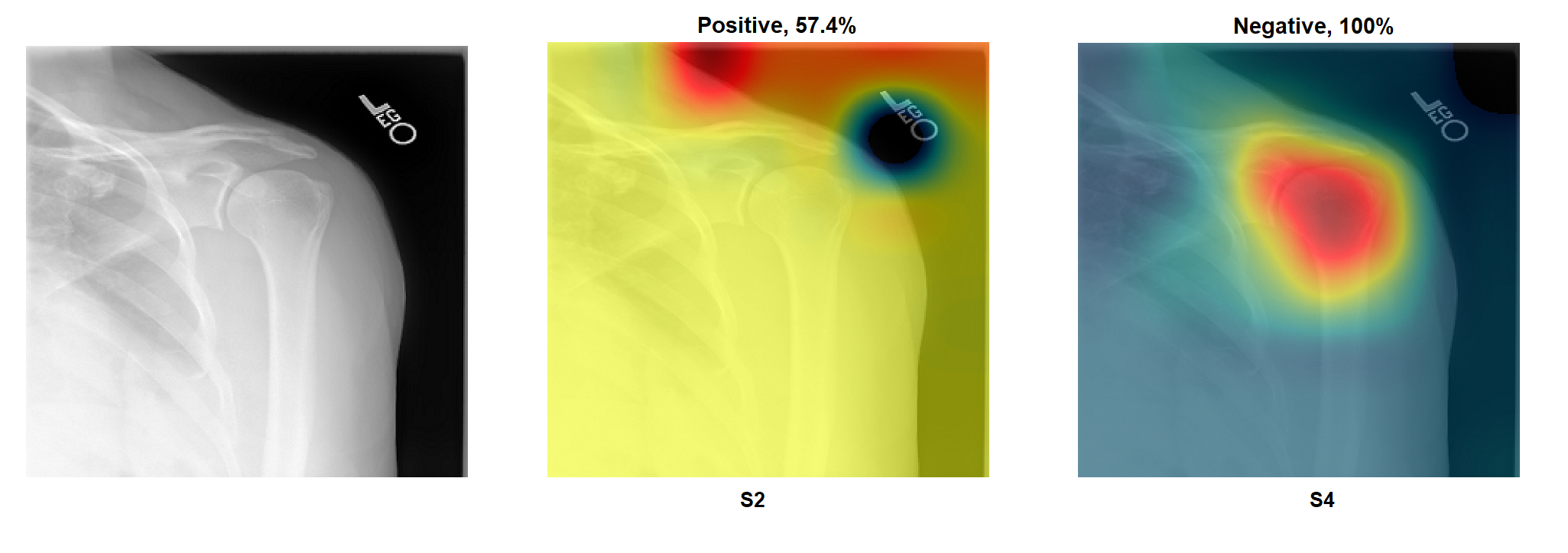

Supplement: S1 Appendix — 1.1 -Grad-CAM and Score- Grad-CAM analyses of Negative shoulder X-ray image. 1.2 -Grad-CAM and Score- Grad-CAM analyses of Negative shoulder X-ray image. 1.3 -Grad-CAM and Score- Grad-CAM analyses of Negative shoulder X-ray image. 1.4 -Grad-CAM and Score- Grad-CAM analyses of Negative shoulder X-ray image. 1.5 -Grad-CAM and Score- Grad-CAM analyses of Negative shoulder X-ray image. 1.6 -Grad-CAM and Score- Grad-CAM analyses of Negative shoulder X-ray image.1.7 -Grad-CAM and Score- Grad-CAM analyses of Negative shoulder X-ray image. (ZIP) [file pone.0299545.s001.zip › App1.1.png]

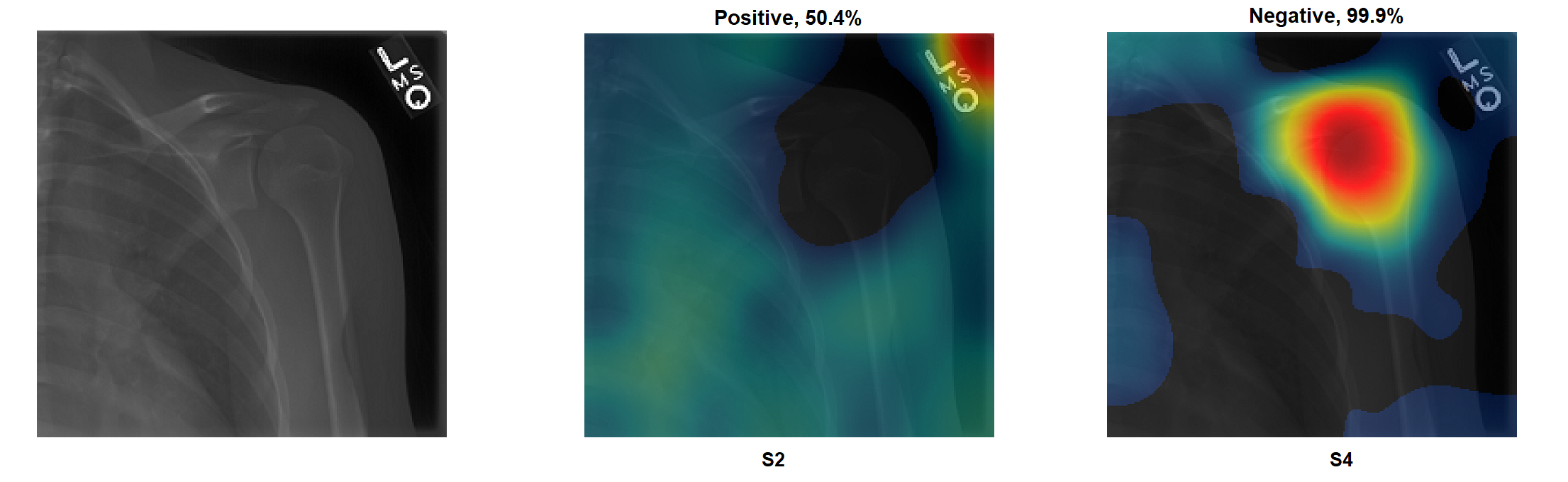

Supplement: S1 Appendix — 1.1 -Grad-CAM and Score- Grad-CAM analyses of Negative shoulder X-ray image. 1.2 -Grad-CAM and Score- Grad-CAM analyses of Negative shoulder X-ray image. 1.3 -Grad-CAM and Score- Grad-CAM analyses of Negative shoulder X-ray image. 1.4 -Grad-CAM and Score- Grad-CAM analyses of Negative shoulder X-ray image. 1.5 -Grad-CAM and Score- Grad-CAM analyses of Negative shoulder X-ray image. 1.6 -Grad-CAM and Score- Grad-CAM analyses of Negative shoulder X-ray image.1.7 -Grad-CAM and Score- Grad-CAM analyses of Negative shoulder X-ray image. (ZIP) [file pone.0299545.s001.zip › App1.2.png]

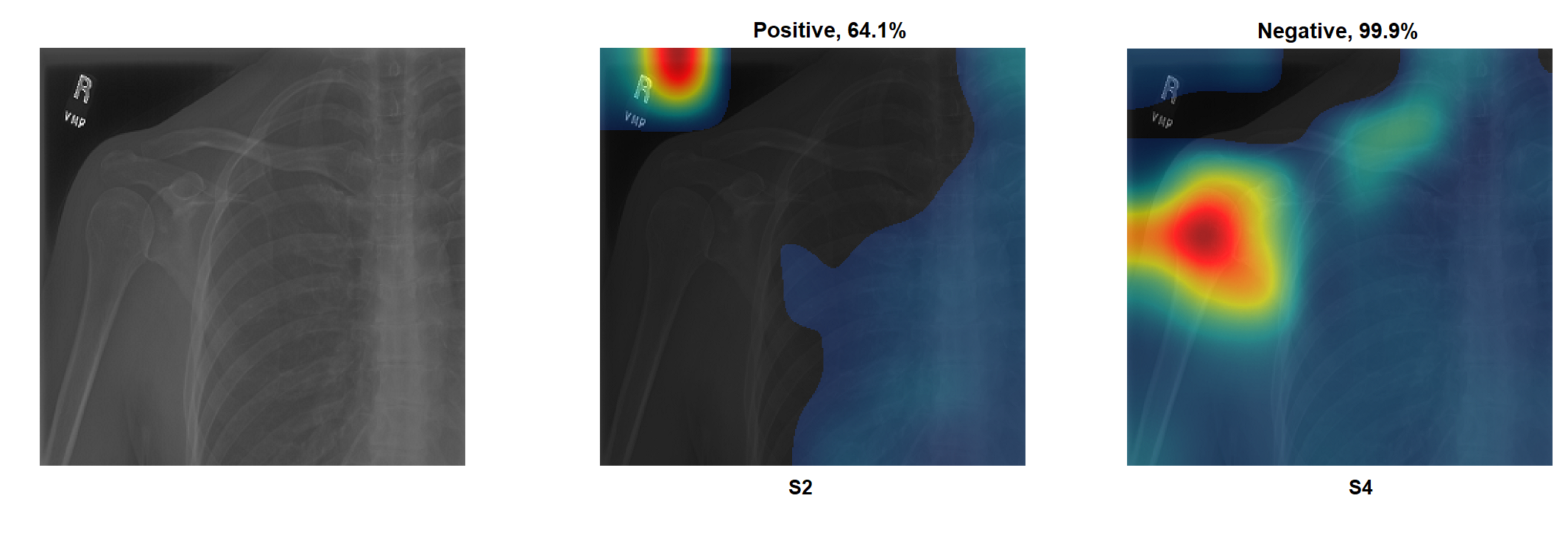

Supplement: S1 Appendix — 1.1 -Grad-CAM and Score- Grad-CAM analyses of Negative shoulder X-ray image. 1.2 -Grad-CAM and Score- Grad-CAM analyses of Negative shoulder X-ray image. 1.3 -Grad-CAM and Score- Grad-CAM analyses of Negative shoulder X-ray image. 1.4 -Grad-CAM and Score- Grad-CAM analyses of Negative shoulder X-ray image. 1.5 -Grad-CAM and Score- Grad-CAM analyses of Negative shoulder X-ray image. 1.6 -Grad-CAM and Score- Grad-CAM analyses of Negative shoulder X-ray image.1.7 -Grad-CAM and Score- Grad-CAM analyses of Negative shoulder X-ray image. (ZIP) [file pone.0299545.s001.zip › App1.3.png]

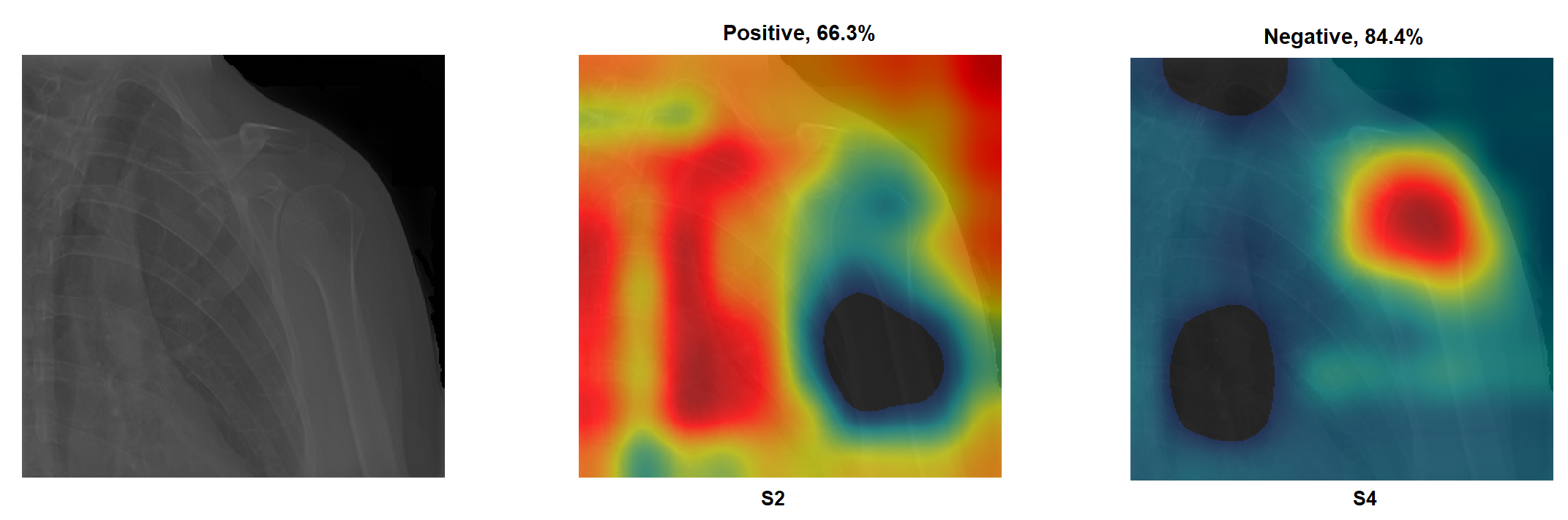

Supplement: S1 Appendix — 1.1 -Grad-CAM and Score- Grad-CAM analyses of Negative shoulder X-ray image. 1.2 -Grad-CAM and Score- Grad-CAM analyses of Negative shoulder X-ray image. 1.3 -Grad-CAM and Score- Grad-CAM analyses of Negative shoulder X-ray image. 1.4 -Grad-CAM and Score- Grad-CAM analyses of Negative shoulder X-ray image. 1.5 -Grad-CAM and Score- Grad-CAM analyses of Negative shoulder X-ray image. 1.6 -Grad-CAM and Score- Grad-CAM analyses of Negative shoulder X-ray image.1.7 -Grad-CAM and Score- Grad-CAM analyses of Negative shoulder X-ray image. (ZIP) [file pone.0299545.s001.zip › App1.4.png]

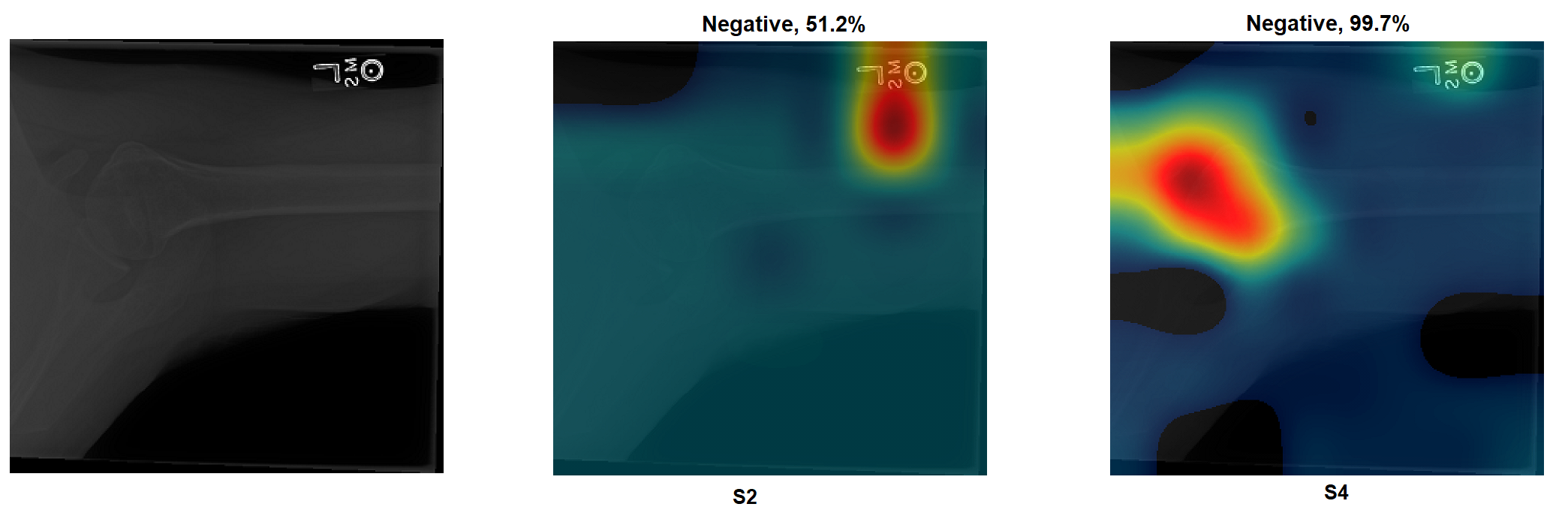

Supplement: S1 Appendix — 1.1 -Grad-CAM and Score- Grad-CAM analyses of Negative shoulder X-ray image. 1.2 -Grad-CAM and Score- Grad-CAM analyses of Negative shoulder X-ray image. 1.3 -Grad-CAM and Score- Grad-CAM analyses of Negative shoulder X-ray image. 1.4 -Grad-CAM and Score- Grad-CAM analyses of Negative shoulder X-ray image. 1.5 -Grad-CAM and Score- Grad-CAM analyses of Negative shoulder X-ray image. 1.6 -Grad-CAM and Score- Grad-CAM analyses of Negative shoulder X-ray image.1.7 -Grad-CAM and Score- Grad-CAM analyses of Negative shoulder X-ray image. (ZIP) [file pone.0299545.s001.zip › App1.5.png]

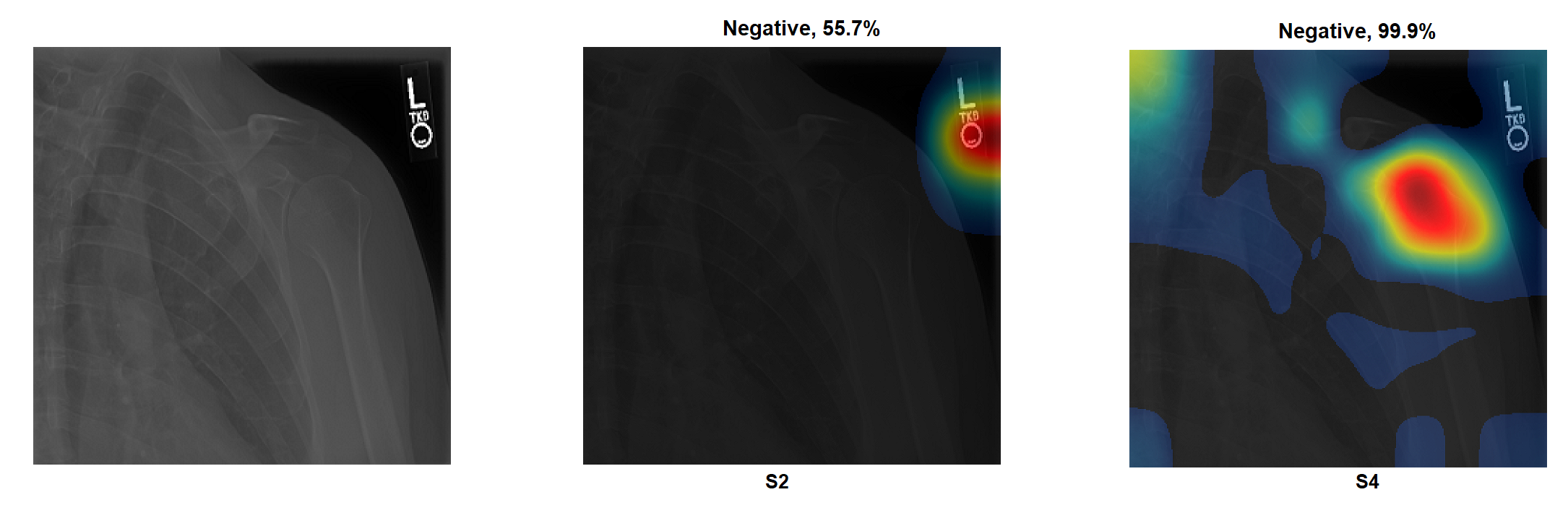

Supplement: S1 Appendix — 1.1 -Grad-CAM and Score- Grad-CAM analyses of Negative shoulder X-ray image. 1.2 -Grad-CAM and Score- Grad-CAM analyses of Negative shoulder X-ray image. 1.3 -Grad-CAM and Score- Grad-CAM analyses of Negative shoulder X-ray image. 1.4 -Grad-CAM and Score- Grad-CAM analyses of Negative shoulder X-ray image. 1.5 -Grad-CAM and Score- Grad-CAM analyses of Negative shoulder X-ray image. 1.6 -Grad-CAM and Score- Grad-CAM analyses of Negative shoulder X-ray image.1.7 -Grad-CAM and Score- Grad-CAM analyses of Negative shoulder X-ray image. (ZIP) [file pone.0299545.s001.zip › App1.6.png]

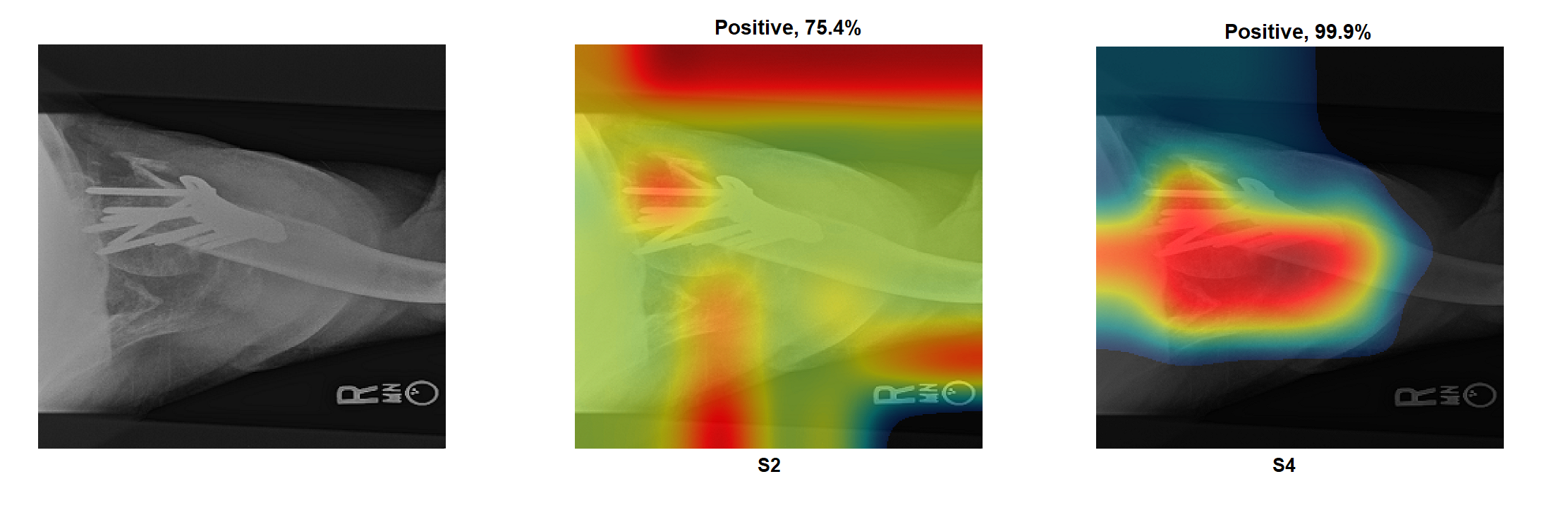

Supplement: S2 Appendix — 2.1 -Grad-CAM and Score- Grad-CAM analyses of Positive shoulder X-ray image. 2.2 -Grad-CAM and Score- Grad-CAM analyses of Positive shoulder X-ray image. 2.3 -Grad-CAM and Score- Grad-CAM analyses of Positive shoulder X-ray image. 2.4 -Grad-CAM and Score- Grad-CAM analyses of Positive shoulder X-ray image. 2.5 -Grad-CAM and Score- Grad-CAM analyses of Positive shoulder X-ray image. 2.6 -Grad-CAM and Score- Grad-CAM analyses of Positive shoulder X-ray image. (ZIP) [file pone.0299545.s002.zip › App2.6.png]

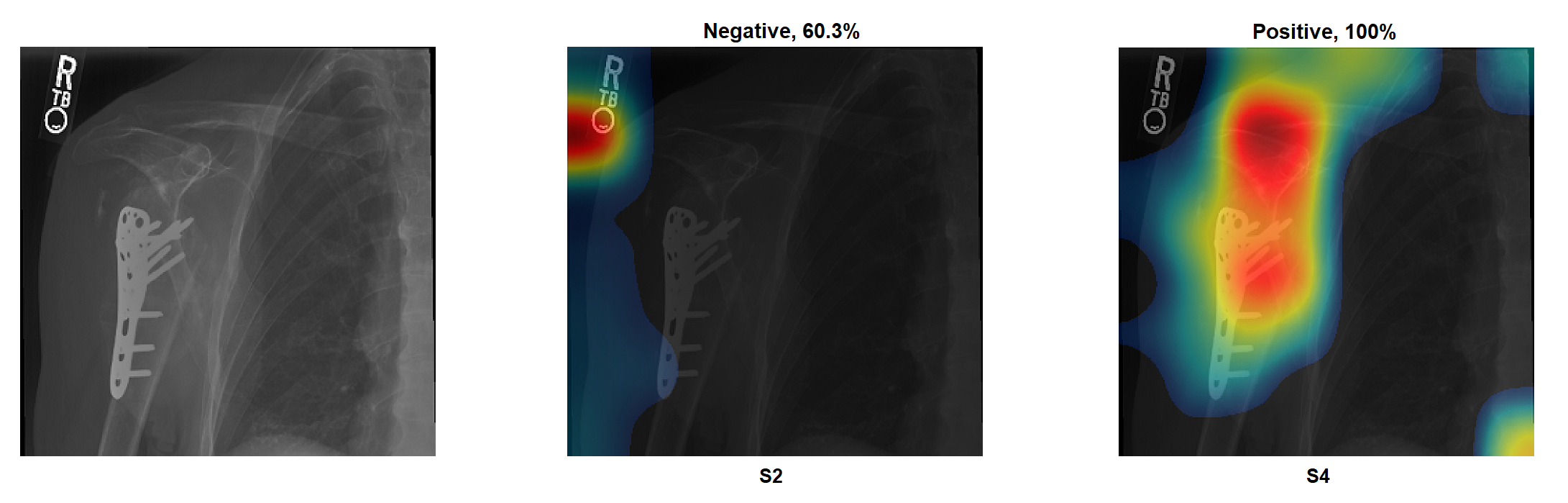

Supplement: S2 Appendix — 2.1 -Grad-CAM and Score- Grad-CAM analyses of Positive shoulder X-ray image. 2.2 -Grad-CAM and Score- Grad-CAM analyses of Positive shoulder X-ray image. 2.3 -Grad-CAM and Score- Grad-CAM analyses of Positive shoulder X-ray image. 2.4 -Grad-CAM and Score- Grad-CAM analyses of Positive shoulder X-ray image. 2.5 -Grad-CAM and Score- Grad-CAM analyses of Positive shoulder X-ray image. 2.6 -Grad-CAM and Score- Grad-CAM analyses of Positive shoulder X-ray image. (ZIP) [file pone.0299545.s002.zip › App2.1.png]

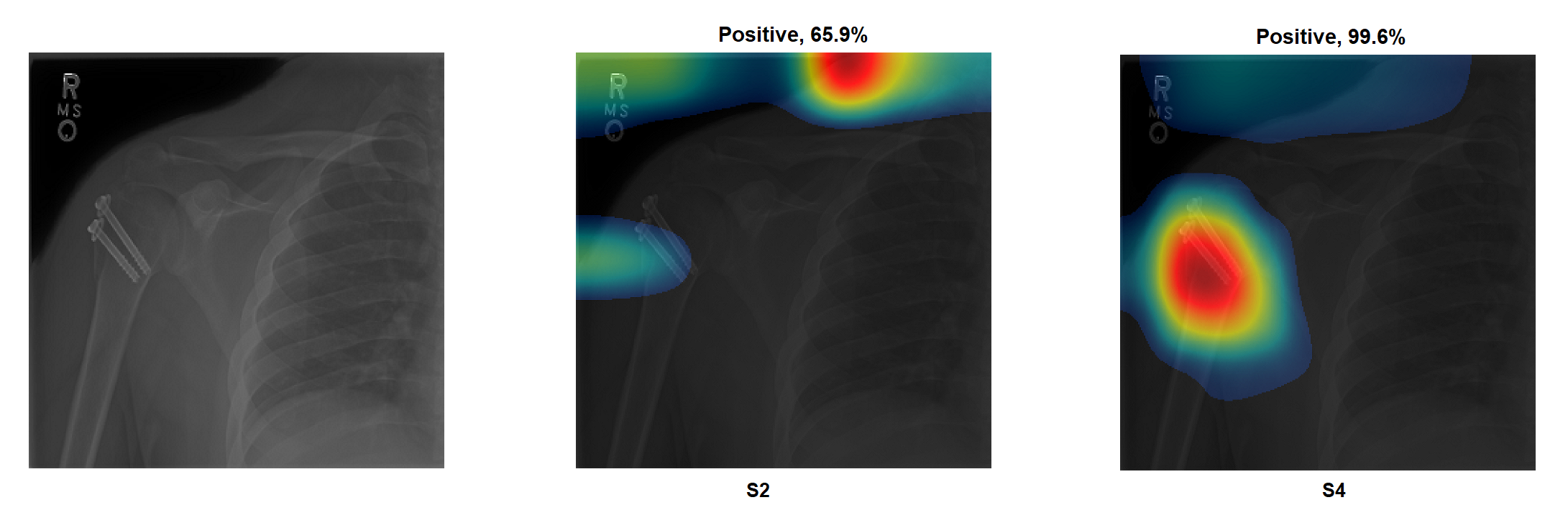

Supplement: S2 Appendix — 2.1 -Grad-CAM and Score- Grad-CAM analyses of Positive shoulder X-ray image. 2.2 -Grad-CAM and Score- Grad-CAM analyses of Positive shoulder X-ray image. 2.3 -Grad-CAM and Score- Grad-CAM analyses of Positive shoulder X-ray image. 2.4 -Grad-CAM and Score- Grad-CAM analyses of Positive shoulder X-ray image. 2.5 -Grad-CAM and Score- Grad-CAM analyses of Positive shoulder X-ray image. 2.6 -Grad-CAM and Score- Grad-CAM analyses of Positive shoulder X-ray image. (ZIP) [file pone.0299545.s002.zip › App2.2.png]

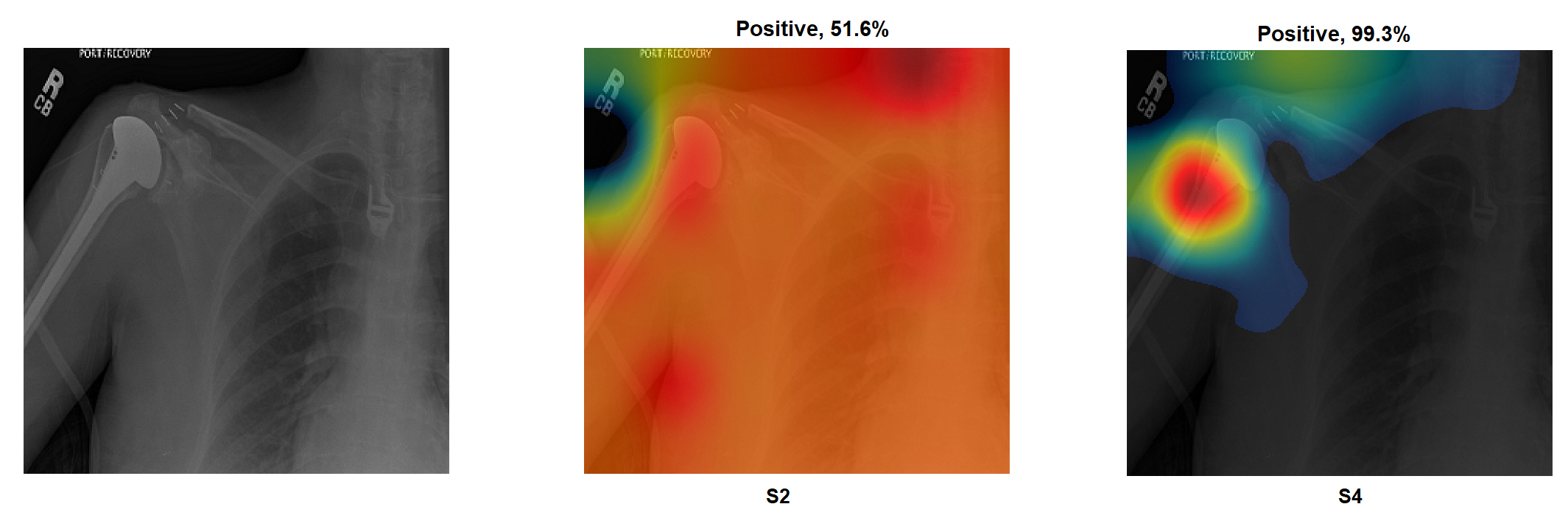

Supplement: S2 Appendix — 2.1 -Grad-CAM and Score- Grad-CAM analyses of Positive shoulder X-ray image. 2.2 -Grad-CAM and Score- Grad-CAM analyses of Positive shoulder X-ray image. 2.3 -Grad-CAM and Score- Grad-CAM analyses of Positive shoulder X-ray image. 2.4 -Grad-CAM and Score- Grad-CAM analyses of Positive shoulder X-ray image. 2.5 -Grad-CAM and Score- Grad-CAM analyses of Positive shoulder X-ray image. 2.6 -Grad-CAM and Score- Grad-CAM analyses of Positive shoulder X-ray image. (ZIP) [file pone.0299545.s002.zip › App2.3.png]

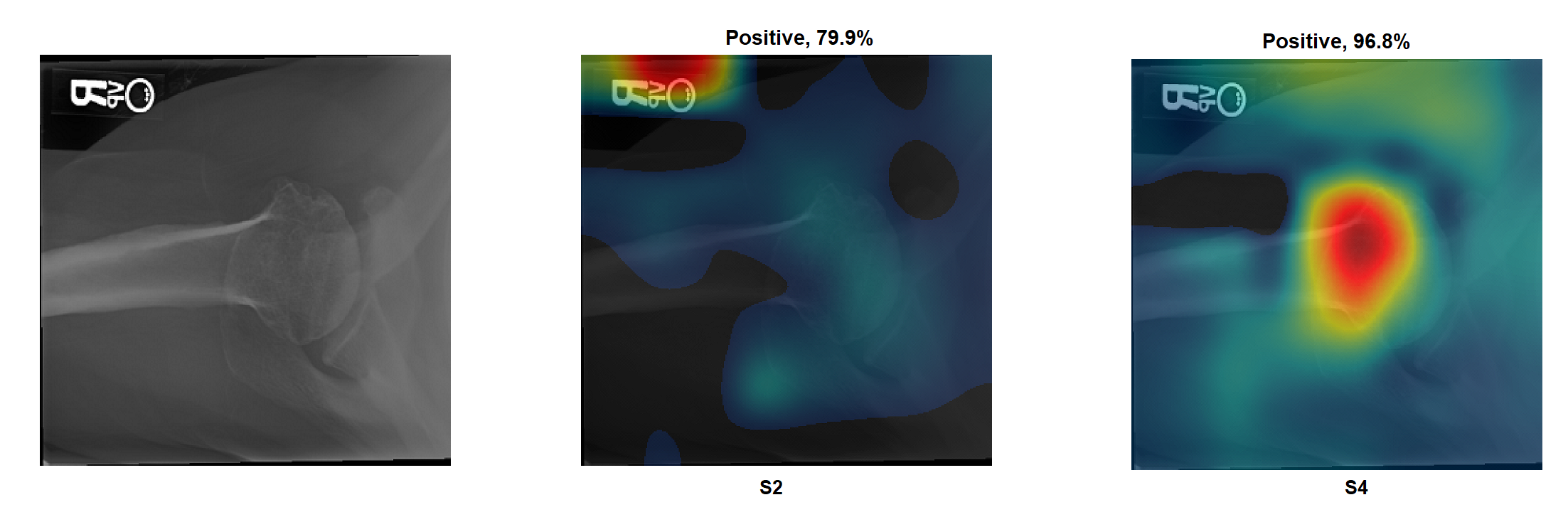

Supplement: S2 Appendix — 2.1 -Grad-CAM and Score- Grad-CAM analyses of Positive shoulder X-ray image. 2.2 -Grad-CAM and Score- Grad-CAM analyses of Positive shoulder X-ray image. 2.3 -Grad-CAM and Score- Grad-CAM analyses of Positive shoulder X-ray image. 2.4 -Grad-CAM and Score- Grad-CAM analyses of Positive shoulder X-ray image. 2.5 -Grad-CAM and Score- Grad-CAM analyses of Positive shoulder X-ray image. 2.6 -Grad-CAM and Score- Grad-CAM analyses of Positive shoulder X-ray image. (ZIP) [file pone.0299545.s002.zip › App2.4.png]

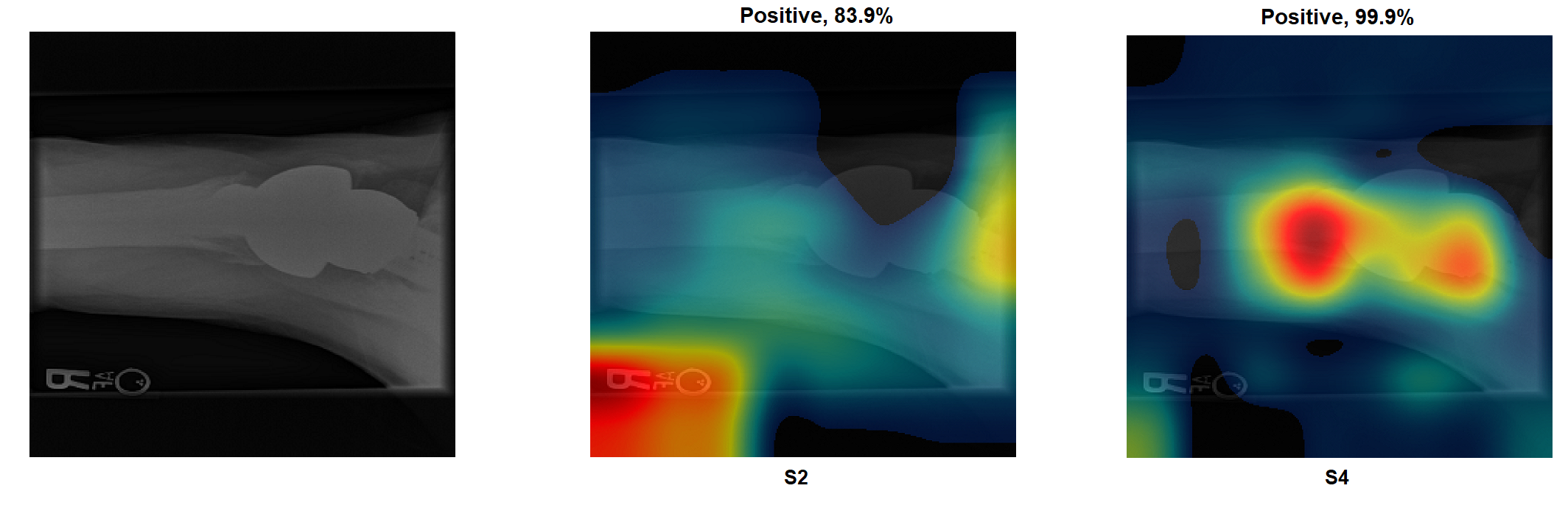

Supplement: S2 Appendix — 2.1 -Grad-CAM and Score- Grad-CAM analyses of Positive shoulder X-ray image. 2.2 -Grad-CAM and Score- Grad-CAM analyses of Positive shoulder X-ray image. 2.3 -Grad-CAM and Score- Grad-CAM analyses of Positive shoulder X-ray image. 2.4 -Grad-CAM and Score- Grad-CAM analyses of Positive shoulder X-ray image. 2.5 -Grad-CAM and Score- Grad-CAM analyses of Positive shoulder X-ray image. 2.6 -Grad-CAM and Score- Grad-CAM analyses of Positive shoulder X-ray image. (ZIP) [file pone.0299545.s002.zip › App2.5.png]
